# MKRN3-mediated ubiquitination of Poly(A)-binding proteins modulates the stability and translation of *GNRH1* mRNA in mammalian puberty

**DOI:** 10.1093/nar/gkab155

**Published:** 2021-03-21

**Authors:** Chuanyin Li, Tianting Han, Qingrun Li, Menghuan Zhang, Rong Guo, Yun Yang, Wenli Lu, Zhengwei Li, Chao Peng, Ping Wu, Xiaoxu Tian, Qinqin Wang, Yuexiang Wang, Vincent Zhou, Ziyan Han, Hecheng Li, Feng Wang, Ronggui Hu

**Affiliations:** State Key Laboratory of Molecular Biology, Shanghai Institute of Biochemistry and Cell Biology, Center for Excellence in Molecular Cell Science, Chinese Academy of Sciences, Shanghai 200031, China; University of Chinese Academy of Sciences, Beijing 100049, China; School of Life Science, Hangzhou Institute for Advanced Study, University of Chinese Academy of Sciences, Hangzhou 310024, China; Cancer Center, Shanghai Tenth People's Hospital, School of Medicine, Tongji University, Shanghai 200031, China; State Key Laboratory of Molecular Biology, Shanghai Institute of Biochemistry and Cell Biology, Center for Excellence in Molecular Cell Science, Chinese Academy of Sciences, Shanghai 200031, China; University of Chinese Academy of Sciences, Beijing 100049, China; State Key Laboratory of Molecular Biology, Shanghai Institute of Biochemistry and Cell Biology, Center for Excellence in Molecular Cell Science, Chinese Academy of Sciences, Shanghai 200031, China; University of Chinese Academy of Sciences, Beijing 100049, China; State Key Laboratory of Molecular Biology, Shanghai Institute of Biochemistry and Cell Biology, Center for Excellence in Molecular Cell Science, Chinese Academy of Sciences, Shanghai 200031, China; University of Chinese Academy of Sciences, Beijing 100049, China; State Key Laboratory of Molecular Biology, Shanghai Institute of Biochemistry and Cell Biology, Center for Excellence in Molecular Cell Science, Chinese Academy of Sciences, Shanghai 200031, China; University of Chinese Academy of Sciences, Beijing 100049, China; State Key Laboratory of Molecular Biology, Shanghai Institute of Biochemistry and Cell Biology, Center for Excellence in Molecular Cell Science, Chinese Academy of Sciences, Shanghai 200031, China; University of Chinese Academy of Sciences, Beijing 100049, China; Department of Juvenile Endocrinology, Ruijin Hospital Affiliated to Shanghai Jiao Tong University, Shanghai 200001, China; State Key Laboratory of Molecular Biology, Shanghai Institute of Biochemistry and Cell Biology, Center for Excellence in Molecular Cell Science, Chinese Academy of Sciences, Shanghai 200031, China; University of Chinese Academy of Sciences, Beijing 100049, China; National Facility for Protein Science in Shanghai, Zhangjiang Lab, Shanghai Advanced Research Institute, Chinese Academy of Science, Shanghai 201210, China; National Facility for Protein Science in Shanghai, Zhangjiang Lab, Shanghai Advanced Research Institute, Chinese Academy of Science, Shanghai 201210, China; National Facility for Protein Science in Shanghai, Zhangjiang Lab, Shanghai Advanced Research Institute, Chinese Academy of Science, Shanghai 201210, China; State Key Laboratory of Molecular Biology, Shanghai Institute of Biochemistry and Cell Biology, Center for Excellence in Molecular Cell Science, Chinese Academy of Sciences, Shanghai 200031, China; University of Chinese Academy of Sciences, Beijing 100049, China; Institute of Nutritional and Health Science, Chinese Academy of Sciences, 320 Yue-yang Road, Shanghai 200031, China; Shao-Hua-Ye M.D. Inc, 416 W Las Tunas Dr Ste 205, San Gabriel, CA 91776, USA; Occidental College, 1600 campus Rd, LA, CA 90041, USA; Department of Thoracic Surgery, Ruijin Hospital Affiliated to Shanghai Jiao Tong University, Shanghai 200001, China; Department of Oral Implantology, Ninth People's Hospital, College of Stomatology, Shanghai Jiao Tong University School of Medicine; National Clinical Research Center for Oral Disease, Shanghai 200001, China; State Key Laboratory of Molecular Biology, Shanghai Institute of Biochemistry and Cell Biology, Center for Excellence in Molecular Cell Science, Chinese Academy of Sciences, Shanghai 200031, China; University of Chinese Academy of Sciences, Beijing 100049, China; School of Life Science, Hangzhou Institute for Advanced Study, University of Chinese Academy of Sciences, Hangzhou 310024, China; Cancer Center, Shanghai Tenth People's Hospital, School of Medicine, Tongji University, Shanghai 200031, China; Guangdong Provincial Key Laboratory of Brain Connectome and Behavior, CAS Key Laboratory of Brain Connectome and Manipulation, the Brain Cognition and Brain Disease, Institute (BCBDI), Shenzhen Institutes of Advanced Technology, Chinese Academy of Sciences, Shenzhen-Hong Kong Institute of Brain Science-Shenzhen Fundamental Research Institutions, Shenzhen 518055, China

## Abstract

The family of Poly(A)-binding proteins (PABPs) regulates the stability and translation of messenger RNAs (mRNAs). Here we reported that the three members of PABPs, including PABPC1, PABPC3 and PABPC4, were identified as novel substrates for MKRN3, whose deletion or loss-of-function mutations were genetically associated with human central precocious puberty (CPP). MKRN3-mediated ubiquitination was found to attenuate the binding of PABPs to the poly(A) tails of mRNA, which led to shortened poly(A) tail-length of *GNRH1* mRNA and compromised the formation of translation initiation complex (TIC). Recently, we have shown that MKRN3 epigenetically regulates the transcription of *GNRH1* through conjugating poly-Ub chains onto methyl-DNA bind protein 3 (MBD3). Therefore, MKRN3-mediated ubiquitin signalling could control both transcriptional and post-transcriptional switches of mammalian puberty initiation. While identifying MKRN3 as a novel tissue-specific translational regulator, our work also provided new mechanistic insights into the etiology of MKRN3 dysfunction-associated human CPP.

## INTRODUCTION

Mammalian puberty is a transition stage between childhood and adulthood, through which mammals attain reproductive capacity and develop secondary gender-specific features (1). The onset of puberty is influenced by complex interactions among genetic, nutritional, environmental and socioeconomic factors (2,3). The anatomic hypothalamic–pituitary–gonadal (HPG) axis regulates both pubertal initiation and development, through a cascade of neuroendocrine events starting from sustained pulsive production and secretion of gonadotropin-releasing hormone (GnRH). With yet incompletely understood mechanisms, humans maintain a largely universal timing for puberty initiation across different races, deviations of which score many clinical conditions including precocious or delayed puberty. An early activation of the HPG axis results in central precocious puberty (CPP), a clinical condition featured by elevated expression and secretion of GnRH in hypothalamus (4,5). In worldwide, CPP is estimated to affection one in ∼5000–10 000 human populations. So far, mutations in many genes were known to be associated with human CPP, particularly those recently reported in the promoter, 5′-UTR (5′-untranslated region) or ORF (open-reading frame) of *MKRN3* gene (2,6–11).

MKRN3 is a maternally imprinted gene and located on the long arm of human chromosome 15 in the region critical for Prader-Willi syndrome (12). MKRN3 belongs to the MAKORIN family including MKRN1, MKRN2 and MKRN3, which usually contain three to four C3H zinc fingers domains, a makorin-specific Cys-His domain and a Ring zinc finger domain that is critical for the activity of RING subfamily E3 ubiquitin ligases (13). MKRNs are expressed in distinct tissue-specific patterns in all mammals (2). Recently, we have shown, for the first time, that MKRN3 knockout mice phenocopy many symptomatic features of human CPP (14), in contrast with the mice deficient of MKRN1 or MKRN2 that do not have phenotypes directly related to puberty, suggesting tissue-specific roles of the MAKORIN family proteins (15). MKRN3 is highly conserved among species, with human and mouse MKRN3 sharing 82% similarity (16). CPP-associated mutations in *MKRN3* gene either reduced expression or caused loss-of-function in the E3 Ub ligase activity of the protein, and MKRN3 was proposed as a‘brake’in regulating puberty initiation in mammals with the underlying mechanism yet incompletely understood. Most recently, we also reported that MKRN3 interacts with, and ubiquitinates MBD3 (methyl-DNA binding protein 3) and epigenetically silences *GNRH1* transcription, through preventing MBD3 from both the recruitment of DNA demethylating Tet2 and the binding to the promoter of *GNRH1* (14). As a result, when CPP-associated mutations occurred in *MKRN3*, such mechanism for epigenetic silencing of *GNRH1* transcription was compromised and GNRH1 expression in hypothalamus was up-regulated to activate the HPG axis and initiate puberty development. It remained incompletely understood whether and how MKRN3 might regulate puberty through targeting other cellular interacting partners or physiological substrates.

PABPs (poly(A)-binding proteins) define a family of proteins that bind to the poly(A) tail of eukaryotic mRNAs and are conserved in species ranging from yeast to human (17). PABPs are multifunctional proteins that regulate many aspects of mRNA homeostasis including mRNA polyadenylation, nonsense-mediated decay (NMD), stress response, controlling mRNA translation initiation, mRNA quality surveillance and so on (17–20). Metazoan PABPC1 were previously shown to promote translation initiation by simultaneously binding to the poly(A) tail and form complex at the 5′-UTR of the mRNAs with many other proteins, including direct interaction with eukaryotic initiation factor 4G (eIF4G) (21,22). Although sharing the core function in promoting mRNA translation and all PABPs have been elegantly demonstrated to be essential for development in vertebrates, their roles seem to be functionally distinct, as the PABPC1-deficient *Xenopus laevis* were embryonical lethal and manifest anterior and posterior phenotypes, which could only be rescued by re-introduced PABPC1 but not by other PABPs family members (23). PABPs, particularly PABPC1, have been recognized as major targets for viruses as viral proteins could cleave or sequester PABPC1 to prevent the formation of complex with eIF4G and shut off host cell translation in favor of the expression viral genes (24). During energy starvation, SIRT1-mediated deacetylation of PABPC1 was found to cause nuclear retention of both PABPC1 protein and mRNA (25). Most recently, PABPC1 was found to be ubiquitinated by RNA-bound MKRN1, which controlled embryonic patterning and germ cell formation (26,27). It is both important and interesting to ask: (i) what are the tissue-specific functions of PABPs? (ii) Whether and how other post-translational modifications would impact the functionality, localization or homeostasis of PABPC1 protein as well as its mRNA? (iii) What pathophysiological significance of these post-translational modifications (PTMs) might confer, by affecting specific mRNA species?

In this work, proteomic analysis was performed and revealed that PABPC1 is one of the most abundant proteins that interacted with MKRN3. Then, PABPC1, PABPC3 and PABPC4 were all found to be substrates for MKRN3, and MKRN3-mediated ubiquitination of PABPs were shown to compromise their binding to the poly (A) tail of target mRNAs, including *GNRH1* mRNA, and affect their stability and translation. Finally, we demonstrated that such mechanism did contribute to post-transcriptional regulation of *GNRH1* expression in both MKRN3-deficient cell and mouse models.

## MATERIALS AND METHODS

### Cell culture and plasmids transfection

HEK293T cells were maintained in Dulbecco's modified Eagle's medium (DMEM), and GT1–7 cells were maintained in DMEM/F12 (1:1), all supplemented with 10% fetal bovine serum (FBS), 50 units/ml penicillin and 50 μg/ml streptomycin (all from Gibco, USA). Primary neurons were isolated from the hypothalamus of wild-type and *Mkrn3^m+/p^^−^* mice at embryonic day 18 and cultured in DMEM/F12 medium (Gibco) supplemented with 10% FBS. On the next day, cells were switched to serum-free NeuroBasal medium (Gibco) supplemented with B-27 supplements (Gibco) and GlutaMAX (Gibco). Cells were maintained at 37°C in a humidified 5% CO_2_ air incubator. All cell lines were routinely tested for mycoplasma contamination, and DNA Transfections were performed using Lipofectamine 2000 reagent (Invitrogen, USA) as instructed by manufacturer.

### Generation of MKRN3 knockout cell lines

*MKRN3* knockout (*MKRN3^−^*^*/*^*^−^*) HEK293T cells were genetically ablated using CRISPR/Cas9 technique and has been used in our previous study (14,28). Briefly, several sequence-guiding RNAs (sgRNAs) for human *MKRN3* gene were designed and the knockdown efficiencies were tested. Cells transfected with sgRNA-expressing vectors were subjected to selection in puromycin (5 μg/ml concentration), with single colonies picked, amplified and subjected to immunoblotting analysis using anti-MKRN3 antibody. Genomic DNAs were extracted using phenol–chloroform extraction method and specific target sequences were then amplified followed by Sanger sequencing to confirm the aimed editions in the genomes of cells.

### Plasmids

The plasmids used in this study were listed in Supplementary Table S1.

### Mice

*Mkrn3* knockout mice were constructed using TALEN-based approach as described before (14), and housed in SPF (specific-pathogen-free) facility. All animal experiments were performed in compliance with the guidance for the care and use of laboratory animals and were approved by the institutional animal research ethics committee of Shanghai institute of Biochemistry and Cell Biology (SIBCB). For genotyping, mouse tail tissues were collected, digested by proteinase K and DNA extracted using phenol-chloroform extraction method. Sequences of the primers used for genotyping were as the following:

Forward primer: 5′-GCCGGGTCTCTTGACGAAGCTGGTCGAGCCATCTC-3′
Reverse primer: 5′-GCCGGCCGGGTCTCGGAGTAGCAGCCGAGCCAATCAGAG-3′
The PCR products were subjected to Sanger sequencing, and *Mkrn3* knockout mice exhibit a 2b deletion.

### Antibodies and Agarose beads

Protein G Agarose beads (16–266) were purchased from Merck Millipore (USA). The antibodies and affinity gels used in this study were listed in Supplementary Table S2.

### Identify MKRN3 interaction proteins by mass spectrometry

Thirty-six 10 cm dishes HEK293T cells were transfected with empty vectors or pCDNA3.0-MKRN3–3xFlag with Lipofectamine 2000 and continued cultured for 48 h. Then cells were lysed in Co-IP buffer (50.0 mM Tris–HCl, 150.0 mM NaCl, 5.0 mM EDTA, and 1.0% NP-40 pH 7.6) supplemented with protease inhibitor cocktail, and incubated with anti-Flag affinity gels overnight at 4°C with or without RNaseA (50 μg/ml). The immunoprecipitates were washed three times with COIP buffer, and eluted with 8M urea dissolved in 100 mM Tris–HCl (pH 8.0), followed by TCEP reduction, CAA alkylation and trypsin digestion. Peptides were separated by the EASY-nLC 1000 system (Thermo Fisher, USA). The column (15 cm in length, 75 μm inner diameter) was packed with ReproSil-Pur C18-AQ 1.9 μm resin (Dr Maisch GmbH). The high resolution data acquisitions were acquired on the Q Exactive HF-X mass spectrometer (Thermo Fisher). Peptides was loaded onto the column and separated with a linear gradient of 5–30% buffer B (ACN with 0.1% formic acid) at a flow rate of 300 nl/min over 36 min. The total time for reversed phase analysis was 45 min. Data was acquired in the data-dependent ‘top15’ mode, in which fifteen most abundant precursor ions were selected with high resolution (120 000 @ *m*/*z* 200) from the full scan (300–1500 *m*/*z*) for HCD fragmentation. Precursor ions with singly charged and charge information unassigned were excluded. Resolution for MS/MS spectra was set to 15 000 @ *m*/*z* 200, target value was 1E5 (AGC control enabled) and isolation window was set to 0.7 *m*/*z*. Normalized collision energy was 30.

MS raw files were analyzed by MaxQuant software, version 1.5.2.8 (29), and peptide lists were searched against the human Uniprot FASTA database (protein items: 92607, 201608). The database search was configured with cysteine carbamidomethylation as a fixed modification and N-terminal acetylation and methionine oxidation as variable modifications. Enzyme specificity was set as C-terminal to arginine and lysine as expected using trypsin as proteases. A maximum of two missed cleavages were allowed. Peptide identification was performed with an initial precursor mass deviation up to 7 ppm and a fragment mass deviation of 20 ppm. The false discovery rate (FDR) was set to 0.01 for protein and peptide levels with a minimum length of seven amino acids for peptides identification. Label-free protein quantitation (LFQ) was performed with a minimum ratio count of 2 (30).

To detect the differentially expressed proteins (DEPs), proteins detected (intensity > 0) in at least three samples were considered. Missing values were imputed with the minimum value across our proteome data. Then, data was normalized based on quantile. The one-sided *t* test (as implemented in R software) was used to assess the DEPs. *P*-value was adjusted by Benjamini & Hochberg method. Proteins with adjust *P*-value ≤0.05 and fold change ≥8 were considered to be up expressed genes.

To further explore the functions of DEPs, GO and KEGG enrichment analysis were performed with ‘clusterProfiler’ package in R (31). Adjusted *P*-value ≤0.05 was considered statistically significant. Network was displayed using Cytoscape (32).

The mass spectrometry proteomics data have been deposited to the ProteomeXchange Consortium via the PRIDE (33,34) partner repository with the dataset identifier PXD022346 (http://www.ebi.ac.uk/pride/archive/projects/PXD022346).

### RNAseq data analysis

The sequencing reads was aligned to *Mus musculus* genomic reference (GRCm38) using HISAT2. Read counts were summarized at the gene level in each sample with HTSeq. In order to calculate RNA half-lives with R, read counts were normalized to the sum of the 18S and 28S rRNA. Genes detected (read count > 0) in at least three samples were considered. Differential expression was determined using DESeq2 (35). Genes with adjust *P*-value ≤0.05 and fold change ≥2 were considered as differentially expressed genes (DEGs). GO enrichment of the identified DEGs was performed with ‘clusterProfiler’ package in R. Volcano plots, barchart were drawn in RStudio with the ggplot2 packages. The generated RNAseq data have been deposited in the Gene Expression Omnibus (GEO, Accession No. GSE160467).

### Co-immunoprecipitation, immunoprecipitation and immunoblotting

For co-immunoprecipitation, tissues/cells were lysed in COIP buffer (50 mM Tris–HCl, 150 mM NaCl, 5 mM EDTA and 1% NP-40 pH 7.6) supplemented with protease inhibitor cocktail (Roche, Switzerland). Then, cell lysates were incubated with indicated antibody and Protein G agarose beads, or affinity gels overnight at 4°C with or without RNaseA (50 μg/ml). For immunoprecipitation, cells were lysed in RIPA buffer [50 mM Tris–HCl, 150 mM NaCl, 5 mM EDTA, 0.1% sodium dodecyl sulfate (SDS), 0.5% sodium deoxycholate and 1% NP-40 pH 7.6] supplemented with protease inhibitor cocktail. Then, tissues/cells lysates were incubated with indicated antibody and Protein G agarose beads, or HA/Flag affinity gels overnight at 4°C. The immunoprecipitates were enriched and denatured at 100°C for 10 min in 2× SDS-PAGE loading buffer. The inputs, immunoprecipitates and other cell lysates were then subjected to SDS-PAGE and transferred to a PVDF membrane (Bio-Rad, USA). The membrane was incubated with the appropriate primary antibodies and secondary antibodies that labeled with HRP, then the signals were visualized using a Tanon 5200 Imaging System (Tanon, China).

### Expression and purification of recombinant proteins

GST- or His6 (His)-tag proteins were expressed in the BL21 *Escherichia coli* cells. After IPTG (isopropyl β-d-thiogalactoside, Sigma, USA) induction, cells were pelleted, lysed and incubated with glutathione or Ni^2+^TA beads to enrich the respective proteins, eluted with 20 mM reduced glutathione (GSH, Sigma) or 250 mM imidazole dissolved in PBS buffer (pH 8.0), then dialyzed in PBS buffer supplemented with 20% glycerol before being aliquoted and preserved at –80°C.

### GST pull-down assay

Purified Gst-MKRNs (MKRN1, MKRN2 or MKRN3) or MKRN3 truncation (20 μg), PABPs-His6 or its truncation (20 μg) and Glutathione Sepharose 4B (GE, USA) were incubated at 4°C overnight in 500 μl of pull-down buffer (20 mM Tris–Cl, 100 mM NaCl, 5 mM MgCl_2_, 1 mM EDTA, 1 mM DTT, 0.5% NP-40 and 10 μg/ml BSA pH 7.5). Then the beads were pelleted and washed five times with pull-down buffer. The recovered beads were denatured at 100°C for 10 min in 2x SDS-PAGE loading buffer and subjected to Coomassie blue staining or immunoblotting analysis.

### Immunofluorescence microscopy

Hela cells were transfected with mCherry tagged MKRN3 (pmCherry-C1-MKRN3) and GFP tagged PABPC1 (pEGFP-C1-PABPC1), then the cells were fixed with 4.0% PFA (paraformaldehyde, Sigma) and staining with DAPI (4′,6-diamidino-2-phenylindole, Sigma) as described previously (36). Images were recorded with microscope BX51 (Olympus, Japan).

### *In vitro* ubiquitination assay

*In vitro* ubiquitination was performed as previously described (36). Briefly, recombinant 200 ng His6-Ub, 50 ng UBA1-His6 (E1), 100 ng E2s-His6, 200 ng Gst-E3, and 200 ng PABPs-His6-Flag were added into *in vitro* ubiquitination buffer (25 mM Tris–Cl, 100 mM NaCl, 1 mM DTT, 5 mM MgCl_2_, pH 7.6, supplemented with 2 mM ATP) with a final reaction volume of 50 μl and incubated at 37°C for 1.5 h. The ubiquitination signal was examined through immunoblotting with anti-Flag antibody.

### Mass spectrometry analysis for ubiquitination sites mapping

*In vitro* ubiquitination assays of MKRN3 and PABPC1 were performed firstly, then the production was subjected to mass spectrometry analysis for ubiquitination sites analysis as described previously (37). Briefly, the sample was dissolved in 8 M urea, 100 mM Tris–Cl (pH 8.5), followed by TCEP reduction, NEM alkylation and trypsin digestion. Peptides were separated by the EASY-nLC system (Thermo Fisher) and analyzed by the Q Exactive mass spectrometer (Thermo Fisher). Protein and ubiquitination analysis were performed with Thermo Proteome Discoverer 2.1 (Thermo Fisher) and searched against Uniprot Human database (http://www.uniprot.org/). The mass spectrometry data have been deposited to the ProteomeXchange Consortium via the PRIDE (33,34) partner repository with the dataset identifier PXD022346 (http://www.ebi.ac.uk/pride/archive/projects/PXD022346).

### Reverse transcription PCR (RT-PCR) and quantitative real-time PCR (qPCR)

Tissues from postnatal day 15 (PND 15) wild-type mice were separated and grinded with liquid nitrogen. Total RNAs were extracted with RNAsimple Total RNA Kit (Tiangen, China) and complementary DNAs (cDNAs) were synthesized using ReverTra^®^ Ace Qpcr RT Master Mix (Toyobo, Japan). Mouse *Gapdh*, *Mkrn3*, *Pabpc1*, *Pabpc3* (also known as *Pabpc6*) and *Pabpc4* were amplified using 2xPCR mixure (Tiangen), and detected by DNA gel electrophoresis. For mRNA half-life experiments, cells were incubated with medium supplemented with 5μg/mL Actinomycin D for 0, 2, 4 or 6 h prior to harvest. Total RNAs were extracted from the indicated cells and cDNAs were synthesized. Quantitative PCR (qPCR) Gene amplifications were performed using SYBR Green (Toyobo) on a 7500 real-time PCR machine (ABI7500, Thermo Fisher, USA), QuantStudio™ 6 Flex (Thermo Fisher) or Roche LightCycler^®^ 96 (Roche, Switzerland), with the relative abundance of each transcript normalized to that of *GAPDH/Renilla* gene, using the 2^ΔΔCt^ method (38). Sequences for the primers used in this study were listed in Supplementary Table S3. Shown in Supplementary Table S4 were the detailed data for three independent biological replicates in the RNA decay experiments, and the original raw data for these qPCR were also provided as supplementary file 1.

### Luciferase reporter assays

The 5′- and 3′-UTR (untranslated region) of human *GNRH1* gene were cloned into PGL3-*miniCMV* vector generating PGL3-*GNRH1*(UTR)-Luc. HEK293T cells were seeded at 0.5 × 10^5^ cells/well in 24-well plates, and transiently transfected with PGL3-*GNRH1*(UTR)-Luc, PRL-TK together with other vectors (MKRN3, PABPC1 or their indicated mutants). After 48 hours transfection, the cells were harvested and lysed with 5× passive buffer and subjected to dual-luciferase reporter assay according to manufacturer's instruction (Promega, USA).

### Enzyme-linked immunosorbent assay (ELISA)

The concentrations of GnRH1 were detected by enzyme-linked immunosorbent assay (ELISA) as previous described (14). Briefly, GT1–7 cells were transfected with indicted plasmids; 48 h later, the complete medium was replaced with serum-free DMEM for 24 h to synchronize the cell cycles, and then replaced with complete medium and continued culture for 24 h. The supernatants were harvested and subjected to GnRH1 (RK-040-02, Phoenix pharmaceuticals, USA) concentration analysis according to the manufacturer's recommendations.

### Poly(U)-pull down assay

Poly(U)-pull down assay was performed as previously described (25). Briefly, cells were lysed in lysis buffer [0.1 M NaCl, 10 mM MgCl_2_, 0.3 M Tris–HCl (pH 7.5), 1 mM DTT, 10 mM ribonucleoside vanadyl complexes, 30 U/ml RNasin ribonuclease inhibitor and 1.0% Triton X-100], then the extracts were centrifuged at 10 000 g for 5 min and the insoluble debris were discarded. Before poly(U)-pull down assay, dry poly(U) agarose (Sigma) was hydrated in 0.1M NaCl and 10 mM Tris–HCl (pH 7.4) at room temperature, then washed in elution buffer [0.1 M NaCl, 10 mM EDTA, 50 mM Tris–HCl (pH 7.4), 0.2% SDS and 25.0% formamide] and high salt binding buffer [0.7 M NaCl, 10 mM EDTA, 50 mM Tris–HCl (pH 7.4), 0.2% lauryl sarcosine and 12.0% formamide], one time each for 5 min at 70°C. Then the pretreated poly(U) agarose was added to cell lysates, rotated for 2 h at 4°C, boiled in 2× SDS-PAGE loading buffer for 10min at 100°C, and subjected to immunoblotting analysis.

### RNA electrophoretic mobility shift assay (EMSA)

The RNA electrophoresis mobility shift assay (EMSA) was performed as previously described (39) with some modifications. RNA probe (A)_30_ was synthesized, and labeled with γ-^32^P at the 3′-end with T4 Polynucleotide Kinase (NEB, USA) and purified by phenol–chloroform method. Recombinant human PABPC1/4 or ubiquitinated PABPC1/4 (purified from *in vivo* ubiquitination or *in vitro* ubiquitination using anti-Flag beads, and eluted with Flag peptide) was incubated with labeled probes in 5× EMSA Binding Buffer (Beyotime, China) at room temperature for 1h, and then the assay was performed on 8% polyacrylamide 0.5× TBE gels to resolve the RNA–protein complexes from the free probes. Gels were then dried and exposed to a phosphor-imager (Kodak, USA) before visualization on a FLA 9000 Fuji scanner (Fujifilm Life Science, USA).

### RNA immunoprecipitation (RNAIP) assay

RNA immunoprecipitation assay was performed as previously described (40). Briefly, 10^7^ cells were collected, and the cells pellet were resuspended with an approximately equal volume of polysome lysis buffer [100 mM KCl, 5 mM MgCl_2_, 10 mM HEPES (pH 7.0), 0.5% NP-40, 1 mM DTT, 400 μM vanadyl ribonucleoside complexes (VRC, NEB, USA)] supplemented with 200 units/ml RNase inhibitors (Invitrogen) and protease inhibitor cocktail (1:100, Roche), incubated on ice for 10 min, centrifuged at 15 000 g for 15 min to clear lysate of large particles, and the supernatant were pe-cleared with pre-swell protein G Agarose beads at 4°C for 60 min. For antibody coating of protein G beads preparation, protein G Agarose beads were pre-swollen in NT2 buffer (50 mM Tris–HCl, 150 mM NaCl, 1 mM MgCl_2_, 0.05% NP-40 pH 7.4) supplemented with 5% BSA to a final ratio of 1:5 for 2 h at 4°C, then PABPC1 antibody was added to the beads slurry and incubated for 6 h at 4°C with tumbling end over end. The antibody-coated beads need to be washed with 1.0 ml of ice-cold NT2 buffer for five times to remove unbound antibody as well as contaminants such as RNases, which may be present in the antibody mixture. After the final wash, resuspend beads in 850 μl of ice-cold NT2 buffer, added 200 units of an RNase inhibitor (5 μl RNase Out), 2 μl VRC (to final concentration of 400 μM), 10 μl of 100 mM DTT and 20 mM EDTA, as well as 100 μl pe-cleared supernatant lysate. At the same time, 100 μl of the mixture were saved as the total cellular mRNA (input). The rest of the mixture was incubated for 4 h at 4°C tumbling end over end, washed five times with 1 ml of ice-cold NT2 buffer supplemented with 0.5 M urea and 0.1% SDS, and then re-suspended the beads in 100 μl of NT2 buffer supplemented with 30 μg of proteinase K (Roche) to release the RNP components. RNAs were isolated from the immunoprecipitated pellet, cDNAs were synthesized, and subjected to RNAIP-qPCR analysis. The specific primers of *Gnrh1* for RNAIP-qPCR analysis were shown in Supplementary Table S3.

### Poly(A) tail-length assay

The Poly(A) tail-length of *gnrh1* mRNA were detected using Poly(A) tail-length assay kit (Invitrogen) according to the manufacturer's instruction. Briefly, total RNAs were prepared from cells, and using poly(A) polymerase adds a limited number of guanosine and inosine residues to the 3′-ends of poly(A)-containing RNAs, then the tailed-RNAs are converted to cDNA through reverse transcription using the newly added G/I tails as the priming sites; PCR amplification products are generated using two primer sets: a gene-specific forward and reverse primer set designed upstream of mouse the polyadenylation site (the 3′-UTR of mouse *gnrh1*) is produced as a control for the gene-of-interest, and the second set of primers uses the gene-specific forward primer and the universal reverse primer provided with the kit to generate a product that includes the poly(A) tails of the gene-of-interest, the *gnrh1* specific primers seen in Supplementary Table S3. The PCR products are separated on 4% agarose gel, and the poly(A) tail-lengths of the *gnrh1* are the sizes of poly(A) PCR-amplified products greater than the sizes of *gnrh1* gene-specific primers PCR-amplified products.

### Statistics

Data were analyzed by two tailed unpaired *t*-test or one-way ANOVA with Bonferroni post-hoc test using GraphPad Prism 7 (GraphPad Software Inc., USA). *P < 0.05 was considered to be of significant difference; **P < 0.01 was considered to be of very significant difference.

## RESULTS

### MKRN3 co-immunoprecipitates with multiple PABP family members

**Figure 1.**
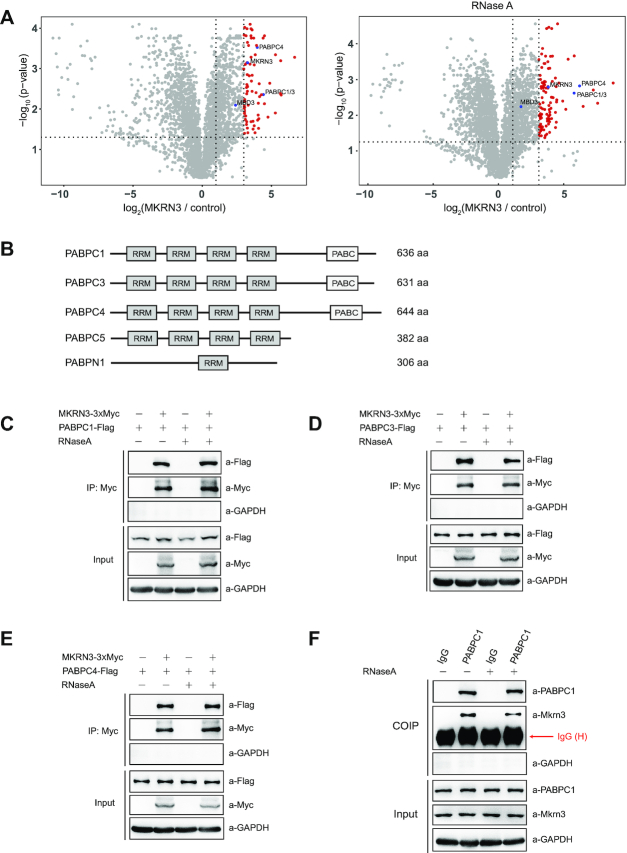
MKRN3 forms complex with poly(A)-binding protein (PABP) family members. (**A**) Identification of MKRN3-interacting proteins through co-immunoprecipitation followed by mass spectrometry analysis. Volcano plots depicting the proteins enrichment from co-immunoprecipitation experiments, and the red circles represent proteins enriched >8-fold by MKRN3 compare to empty vectors. HEK293T cells were ectopically expressing 3xFlag-tagged MKRN3 or Flag tagged empty vectors. Forty-eight hours later, cells lysates treated with or without RNaseA (50 μg/ml), were incubated with anti-Flag affinity gels, and the co-immunoprecipitates were subjected to trypsin digestion followed by mass spectrometry analysis. MKRN3, PABP family members including PABPC1, PABPC3 and PABPC4, and previously reported MKRN3 interacting protein MBD3 indicated in blue colour. Three samples each group. (**B**) Domain structures of the PABP family members, including PABPN1, PABPC1, PABPC3 PABPC4 and PABPC5, which commonly share RNA-binding RRM (RNA recognition motif). PABC, poly(A)-binding protein C-terminal domain. (C–F) 3XMyc tagged MKRN3 protein was co-immunoprecipitated with Flag-tagged PABPC1 (**C**), PABPC3 (**D**) or PABPC4 (**E**) when co-expressed in HEK293T cells. Cell lysates treated with or without RNaseA (50 μg/ml) were subjected to co-immunoprecipitation assay using anti-Myc antibody, followed by immunoblotting with anti-Myc or anti-Flag antibody. (**F**) Endogenous Mkrn3 and PABPC1 formed a complex in the hypothalamus of wild-type mouse. Endogenous PABPC1 proteins of hypothalamic lysates from wild-type male mice at postnatal day 15 treated with or without RNaseA were immunoprecipitated using anti-PABPC1, followed by immunoblotting with indicated antibodies.

MKRN3, an E3 Ub ligase, plays critical role in regulating puberty in mammals. To identify the potential interacting partners and substrates for MKRN3, 3xFlag-tagged MKRN3 was transfected into HEK293T cells, and used as the bait to co-immunoprecipitate (co-IP) with proteins that could form complex with MKRN3. The co-immunoprecipitates were enriched with anti-Flag affinity gels in the presence or absence of RNaseA, resolved on SDS-PAGE gel, and visualized by Commassie Blue staining (Supplementary Figure S1A). Subsequent mass spectrometry analysis revealed a list of proteins that were present in the MKRN3 or control groups (Supplementary Table S5). As shown in Figure 1A and Supplementary Figure S1B, PABP family members including PABPC1, PABPC3 and PABPC4, which shared a common signature PABC (poly(A)-binding protein C-terminal) domain and another four highly conserved RRM (RNA recognition motif) domains (Figure 1B), emerged as the hits of highest confidence, regardless of RNaseA treatment, suggesting that mRNAs were not involved in the interaction between MKRN3 and PABPCs. Individual co-IP tests indicated that the ectopically expressed MKRN3 could form complex with exogenously expressed PABPC1, PABPC3, PABPC4, PABPC5, but not PABPN1 in HEK 293T cells (Figure 1C–E, Supplementary Figure S1C and S1D). Further fluorescence microscopy analysis indicated that GFP tagged MKRN3 and mCherry tagged PABPC1 did co-localize in both cytoplasm and nuclear of Hela cells (Supplementary Figure S1E).

Gene expression profiling analyses performed with wild-type mice aged at postnatal day 15 (PND 15) indicated that *Mkrn3* was mainly expressed in mouse brain and reproductive system including testis and ovary, while *Pabpc3* (also known as *Pabpc6*) was mainly expressed in testis, spleen and lung; *Pabpc1* and *Pabpc4* were expressed in all detected tissues (Supplementary Figure S1F). In lysates prepared with hypothalamus of male mice at postnatal day 15, endogenous Mkrn3 and PABPC1 proteins were co-immunoprecipitated using anti-PABPC1 antibody, regardless of RNaseA treatment (Figure 1F).

These data clearly suggested that MKRN3 could form complex with several members of the PABP family proteins both in cells and tissues that expressing them.

### MKRN3 directly interacts with the C-terminal regions of multiple PABP proteins

**Figure 2.**
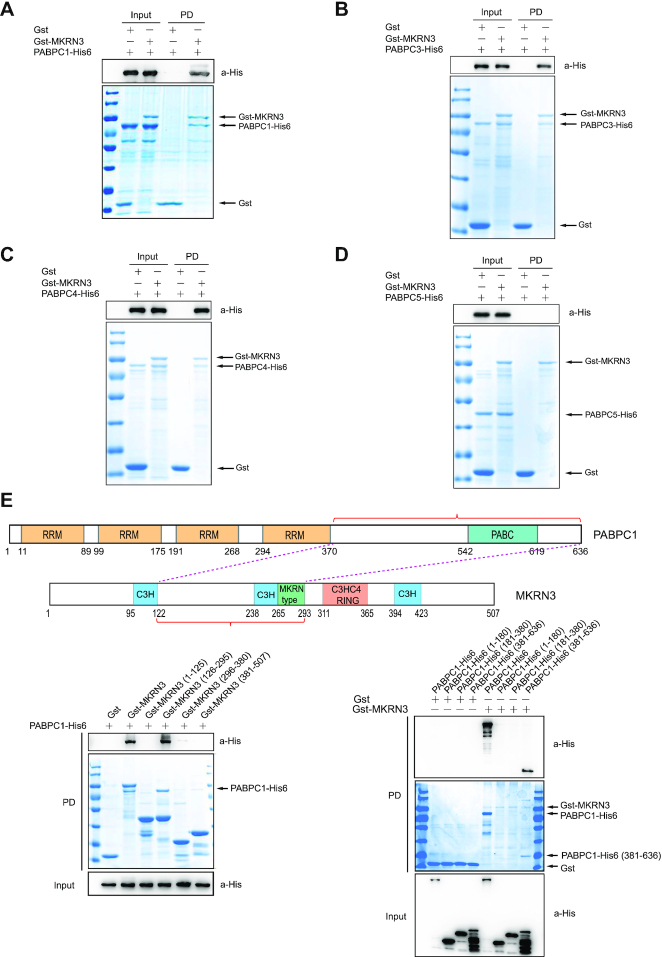
MKRN3 directly interacts with the PABC domain of PABP family members. (**A–D**) Recombinant MKRN3 directly interacted with PABPC1 (A), PABPC3 (B), or PABPC4 (C), but not PABPC5 (D), as revealed by GST pull-down assay. GST pull-down assays were performed with recombinant Gst-tagged MKRN3 and His6-tagged PABPC1, PABPC3, PABPC4 or PABPC5. The recovered Gst tagged proteins were resolved in SDS-PAGE and visualized with Commassie Blue gel staining, while the PABP proteins were detected by immunoblotting with anti-His antibody. PD, GST pull-down. (**E**) The PABC domain of PABPC1 directly interacted with the middle region (126–295aa) of MKRN3. The residue numbers were denoted underneath each schematic structural region of the proteins. GST pull-down assays were performed with recombinant Gst-MKRN3 or its fragments, and PABPC1-His6 or its fragments. PD, GST pull-down.

We went on to test whether MKRN3 might directly interact with PABP family members. GST pull-down assays performed with recombinant Gst-tagged MKRN3 and His6-tagged PABPs (PABPC1, PABPC3, PABPC4 or PABPC5-His6) indicated that MKRN3 could directly interact with PABPC1, PABPC3, PABPC4, but not PABPC5 (Figure 2A–D). MKRN3 belongs to the MAKORIN family members that including MKRN1, MKRN2 and MKRN3, and they are evolutionarily conserved. GST pull-down assays indicated that MKRN1 and MKRN3, but not MKRN2, directly interacted with PABPC1 (Supplementary Figure S2A). Further mapping analysis indicated that the C-terminal regions of PABPC1 interacted with the full length MKRN3, and the middle region (126–295aa) of MKRN3 were directly interacted with the full length PABPC1 (Figure 2E). To further validate this result, the middle region (126–295aa) of MKRN3 was deleted, and found that this mutant lost the ability to interact with PABPC1 (Supplementary Figure S2B). Then, GST pull-down assays were carried out with recombinant Gst-MKRN3 and PABPC1-His6, PABPC1 (381–636)-His6, PABPC5-His6 or the indicated two swapped His6-tagged proteins. PABPC1 with C-terminal region of PABPC5 did not bind to MKRN3, while PABPC5 with the C-terminal part of PABPC1 gain the ability to bind MKRN3 (Supplementary Figure S2C). All these data suggested that the middle region (126–295aa) of MKRN3 directly interacted with the C-terminal region of PABPC1.

### MKRN3 ubiquitinates the PABP proteins that contain PABC domain, which can be impaired by disease-associated mutations in *MKRN3*

**Figure 3.**
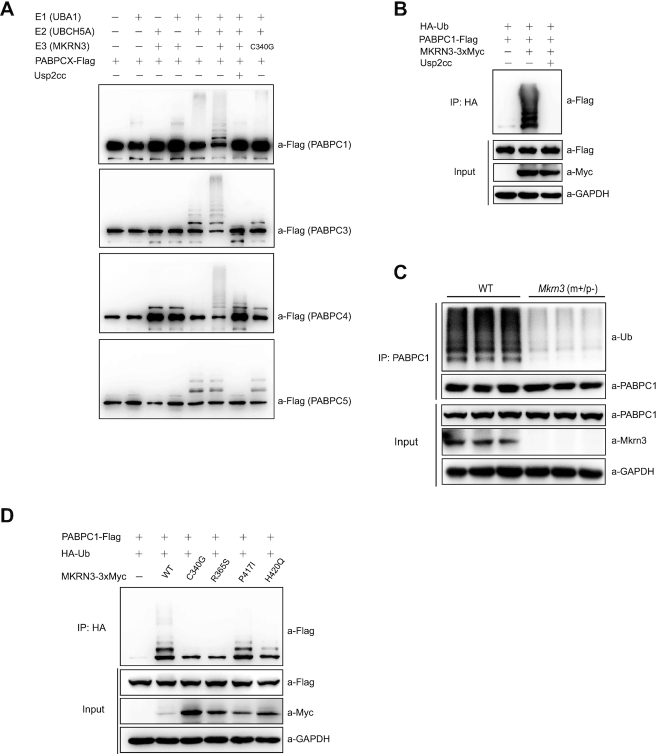
MKRN3 ubiquitinates PABP family members that contain PABC domain and CPP-associated mutations of MKRN3 comprised this ubiquitination. (**A**) MKRN3 ubiquitinated PABPC1, PABPC3 and PABPC4, but not PABPC5 *in vitro*. An *in vitro* ubiquitination assay was carried out using the recombinant Flag tagged PABPC1, PABPC3, PABPC4 or PABPC5, His6-tagged UBA1 (E1) and UBCH5A (E2), and Gst-tagged MKRN3 (E3) or the E3 ligase dead mutant MKRN3 (C340G), together with the indicated components. Usp2cc, the catalytic core of human deubiquitinase Usp2; PABPCX represents PABPC1, PABPC3, PABPC4 or PABPC5. (**B**) Flag-tagged PABPC1 was efficiently ubiquitinated by 3XMyc-tagged MKRN3. *MKRN3^−^^/^^−^* HEK293T cells were co-transfected with indicated plasmids, and cell lysates were immunoprecipitated with anti-HA affinity gels, treated with Usp2cc or not, followed by immunoblotting with anti-Flag antibody to detect ubiquitinated PABPC1. (**C**) The ubiquitination of PABPC1 was reduced in *Mkrn3^m+/p^^−^* mouse hypothalamus at postnatal day 15, compared to those from the age-matched wild-type littermates (*n* = 3). Endogenous PABPC1 proteins of hypothalamic lysates from wild-type or *Mkrn3^m+/p^^−^* mice were immunoprecipitated using anti-PABPC1 antibody, followed by immunoblotting with anti-Ub or other indicated antibodies. (**D**) CPP-associated mutations of *MKRN3* compromised its E3 ligase activity towards PABPC1 in *MKRN3^−^^/^^−^* HEK293T cells. Cells were transfected with HA-tagged Ub, Flag-tagged PABPC1, and 3xMyc-tagged wild-type MKRN3 or the indicated mutants (CPP-associated mutations, C340G, R365S, P417I and H420C). Cell lysates were immunoprecipitated with anti-HA affinity gels, followed by immunoblotting with anti-Flag antibody to detect ubiquitinated PABPC1 and other antibodies indicated.

Ubiquitination assays were carried out to check whether the PABP proteins were merely its interacting partners or a group of novel substrates for E3 ligase MKRN3. Firstly, as shown in Figure 3A, recombinant MKRN3 was able to conjugate poly-Ub chains onto PABPC1, PABPC3, PABPC4, but not PABPC5, which was consistent with the results of GST pull-down assays (Figure 2A–D). It was interesting to note that the conjugating enzymes UBCH5 family members, including UCH5A, UBCH5B and UBCH5C, but not UBCH7, UBC7 or UBC13 could support MKRN3-mediated ubiquitination of PABPC1 (Supplementary Figure S3A). Meanwhile, it was clear that MKRN1, but not MKRN2, was also able to mediate the ubiquitination of PABPC1 (Supplementary Figure S3B and C).

Consistently, ectopically expressed MKRN3 increased the ubiquitination of PABPC1 in *MKRN3^−^^/–^*HEK293T cells which could be removed by Usp2cc, the catalytic core of human deubiquitinase USP2 (Figure 3B). Similar results were also obtained with PABPC3 or PABPC4 (Supplementary Figure S3D and E). Further study revealed that endogenous PABPC1 did undergo much less ubiquitination in the hypothalamus of *Mkrn3^m+/p^^−^* mice compared to that of age-matched wild-type littermates at postnatal day 15 (Figure 3C). Therefore, PABPC1, PABPC3 and PABPC4, were identified as physiological substrates for MKRN3 in mammals.

Previously, we have shown that the CPP-associated mutations in human *MKRN3* did compromise the E3 Ub ligase activity of MKRN3 towards MBD3 and its autoubiquitination (14). As shown in Figure 3D and Supplementary Figure S3F, the CPP-associated MKRN3 mutants impaired their ability in conjugating poly-Ub chains to PABPC1 and PABPC4, with the MKRN3 (P417I) mutant as an exception. Considering the authenticity of PABPC1, PABPC3 and PABPC4 as substrates for MKRN3, these data suggested the possibility that CPP-associated mutations could also contribute to the disease phenotypes through affecting the functionality of pathways regulating RNA homeostasis.

### MKRN3 mediates non-proteolytic ubiquitination of PABPC1 at multiple sites

**Figure 4.**
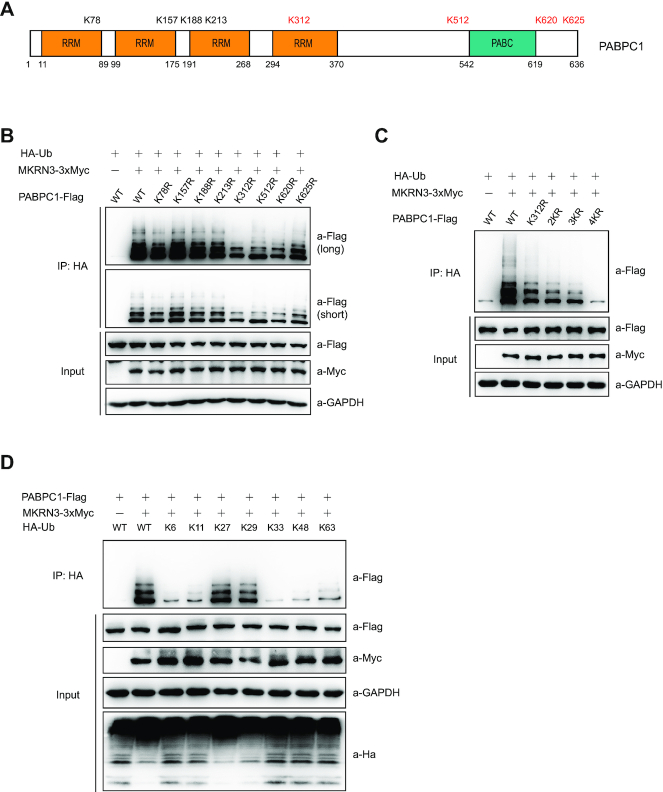
MKRN3 mediates non-proteolytic ubiquitination of PABPC1 at multiple sites. (**A**) Schematic distribution of the eight sites (Lys residues) for MKRN3-mediated ubiquitination on human PABPC1 identified by mass spectrometry analysis. PABPC1 proteins were recovered from the *in vitro* ubiquitination assay and subjected to trypsin digestion, followed by mass spectrometry analysis to map the ubiquitination sites. (**B**) Four Lys residues (K312, K512, K620 and K625, shown red colour in A) were shown to be the major sites for MKRN3-mediated ubiquitination of PABPC1. Lysates of *MKRN3^−^^/-^*HEK293T cells ectopically expressing HA tagged Ub, 3XMyc tagged MKRN3, and Flag tagged PABPC1 or the indicated K-to-R mutants. Cell lysates were immunoprecipitated with anti-HA affinity gels, followed by immunoblotting analysis using anti-Flag to detect the ubiquitinated PABPC1 and other antibodies indicated. (**C**) Simultaneous four K-to-R mutation at the major ubiquitination sites almost totally abolished the ubiquitination of PABPC1 mediated by MKRN3. *MKRN3^−^^/-^*HEK293T cells were ectopically expressing HA tagged Ub, 3XMyc tagged MKRN3 and Flag-tagged PABPC1 or the mutants bearing the indicated K-to-R mutations. Cell lysates were immunoprecipitated with anti-HA affinity gels, followed by immunoblotting using anti-Flag to detect the ubiquitinated PABPC1. 2KR, K312–512R; 3KR, K312–512-620R; 4KR, K312–512-620–625R. (**D**) MKRN3 ubiquitinated PABPC1 with non-proteolytic K27 and k29 ubiquitin linkages. *MKRN3^−^^/–^*HEK293T cells were ectopically co-expressing 3xMyc-tagged MKRN3, Flag-tagged PABPC1 and HA tagged Ub (WT, or mutated at Lys6, Lys11, Lys27, Lys29, Lys33, Lys48 or Lys63 only) in the indicated combinations. Cell lysates were were immunoprecipitated with anti-HA affinity gels, followed by immunoblotting using anti-Flag to detect the ubiquitinated PABPC1 and other antibodies indicated.

To further characterize MKRN3-mediated ubiquitination of PABPC1, PABPC1 was recovered from the above *in vivo* ubiquitination assay (Figure 3A) and subjected to mass spectrometry analysis. As shown in Figure 4A and Supplementary Table S6, eight Lys (K) residues of PABPC1 (K78, K157, K188, K213, K312, K512, K620 and K625) were identified as ubiquitination sites mediated by recombinant MKRN3 *in vitro*. *In vivo* ubiquitination assays indicated that four Lys residues (312, 512, 620 and 625, shown red colour in Figure 4A) of PABPC1 turned out to be the major sites for MKRN3-mediated ubiquitination in *MKRN3^−^^/–^* HEK293T cells, as the PABPC1 mutants that bore these individual Lys-to-Arg (K-to-R) substitutions showed much attenuated ubiquitination levels (Figure 4B). Furthermore, the mutant bearing simultaneous K-to-R substitutions (4KR) at all these four Lys residues almost totally abolished MKRN3-mediated ubiquitination on PABPC1 (Figure 4C). Of note, K312 was located in the fourth RRM domain in PABPC1, while K512, K620 and K625 resided in the regions flanking the PABC domain. These four ubiquitination sites were conserved in human PABP family members that contain the PABC domain, and also conserved in different origins (human, mouse and rat) according to sequence alignment analysis (Supplementary Figure S4A), suggesting that MKRN3-mediated ubiquitination is likely to occur on the other PABP orthologs as well. Furthermore, MKRN3 seemed to have no detectable effect on the stability of PABPC1 protein (Supplementary Figure S4B and C), indicating a non-proteolytic function of the ubiquitination event. Indeed, when ubiquitination assay was performed with *MKRN3^−^^/-^* HEK293T cells ectopically expressing 3xMyc-tagged MKRN3 (MKRN3–3xMyc), Flag-tagged PABPC1 and HA-tagged wild-type Ub, or Ub mutants that bore K-to-R mutations at all Lys residues except one intact Lys only at the indicated residue (K6, K11, K27, K29, K33, K48, K63), MKRN3 seemed to conjugate poly-Ub chains on PABPC1 predominantly in K27 and K29 linkages (Figure 4D). It was thus clear that MKRN3 mediated the ubiquitination of PABPC1 with poly-Ub chains in non-proteolytic Lys (K) linkages.

### The ubiquitination of PABPs by MKRN3 destabilizes *GNRH1* mRNA and down-regulates its expression in GT1-7 cells and mouse hypothalamus

**Figure 5.**
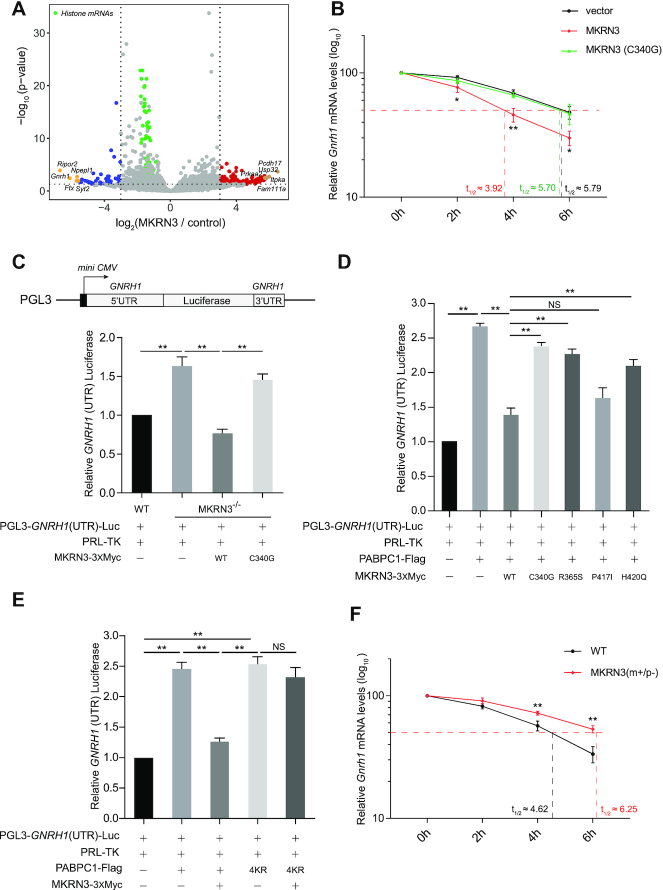
The ubiquitination of PABPs by MKRN3 attenuates the stability of *GNRH1* mRNA. (**A**) Volcano plots depicting MKRN3 up-regulated and down-regulated genes detected by RNA-seq. Hypothalamus-derived GT1–7 cells were transfected with empty or MKRN3 expressing vectors, and RNA-seq were performed 48 h later, three samples each group. The red circles represent genes that are up-regulated by MKRN3 more than eight times, the blue circles represent genes that are down-regulated by MKRN3 more than 8 times and the green circles represent histone mRNAs. The yellow circles represent the top five up-regulated and down-regulated genes. (**B**) MKRN3 destabilized *Gnrh1* mRNA in GT1–7 cells. Cells were transfected with empty vector, or vector expressing MKRN3 or MKRN3 (C340G), and treated with Actinomycin D (5 μg/mL) to block de novo transcription of *Gnrh1* at different time points (0, 2, 4 or 6 h) after 36 h transfection. Total mRNAs were extracted and subjected to quantitative PCR (qPCR) with *Gnrh1* signals normalized to that of *Gapdh*. Each sample was normalized to 100% at time zero, and the half-lives of *Gnrh1* mRNA (*t*_1/2_) were calculated. Data were presented as mean ± SD, three independent experiments. MKRN3 versus vector, *P < 0.05, significant difference; **P < 0.01, very significant difference. (**C**) Luciferase reporter assay to detect the relative activities of human *GNRH1* (UTR). A luciferase reporter that contains 3′- and 5′-UTR (untranslated region) of human *GNRH1* were constructed, named as PGL3-*GNRH1*(UTR)-Luc, and its activities were detected in wild-type or *MKRN3^−^*^*/−*^HEK293T cells that ectopically expressing empty vector, MKRN3 or MKRN3 (C340G). Data were presented as mean ± SD, one-way ANOVA with Bonferroni post-hoc test. **P < 0.01, very significant difference, three independent experiments. (**D**) CPP-associated mutations compromised the suppressive effect of MKRN3 on *GNRH1* (UTR) luciferase activity. *MKRN3^−^^/–^* HEK293T cells were co-transfected with plasmids encoding *GNRH1* (UTR) luciferase, PABPC1, wild-type MKRN3 or the indicated mutants. Data were presented as Mean ± SD, one-way ANOVA with Bonferroni post-hoc test. **P < 0.01, very significant difference; NS, no significant difference, three independent experiments. (**E**) MKRN3-mediated ubiquitination impaired PABPC1-activated *GNRH1* (UTR) luciferase activity. *MKRN3^−^^/^^−^* HEK293T cells were co-transfected with vectors encoding *GNRH1* (UTR) luciferase, MKRN3 and PABPC1 or PABPC1_4KR_. Data were presented as mean ± SD, one-way ANOVA with Bonferroni post-hoc test. **P < 0.01, very significant difference; NS, no significant difference, three independent experiments. (**F**) *Gnrh1* mRNA was more stable in the neurons of *Mkrn3^m+/p^^−^* mouse than that of wild-type. Neurons were isolated from the hypothalamus of wild-type or *Mkrn3*^m+/p−^ fetal male mice at embryonic day 18, subsequently cultured for 15 days, and treated with Actinomycin D (5 μg/ml) at different time points (0, 2, 4 or 6h). Total mRNAs were extracted and subjected to qPCR with *Gnrh1* signals normalized to that of *Gapdh*. Each sample was normalized to 100% at time zero, and the half-lives of *Gnrh1* mRNA (*t*_1/2_) were calculated. Data were presented as mean ± SD, three independent experiments. **P < 0.01, very significant difference.

PABPC1 commonly binds to the poly(A) tail of mRNA, affects their stability and is recruited into a complex that regulate mRNA translation (41). It was interesting to ask whether the ubiquitination of PABP family proteins have any effect on the function and stability of target mRNAs. To further address this issue, mouse hypothalamus-derived GT1–7 neuron cells that don’t express endogenous Mkrn3 protein was transfected with either empty vector or pCDNA3.0–MKRN3–3xFlag, and subjected to RNA-seq analysis. As shown in Figure 5A and Supplementary Table S7, 354 genes appeared to be up-regulated by MKRN3, while 395 genes were down-regulated by MKRN3 (only genes with adjusted *P*-value ≤0.05 and fold changes ≥2 were considered as DEGs (differentially expressed genes)). Gene Ontology (GO) enrichment of these DEGs indicated that MKRN3 seemed to substantially impact neuronal, reproductive and development-related signalling pathways (Supplementary Figure S5A). As shown in Figure 5A, *Pcdh17, Usp32, Itpka, Prkaa2* and *Fam111a* were the top five genes among the 131 hits that up-regulated by MKRN3 over eight times (blue colour), and *Ripor2, Gnrh1, Ftx, Npepl1* and *Syt2* were the top five genes among 44 hits that were down-regulated by MKRN3 over eight times (red colour), which were each subjected to further validation tests. Consistent results from qPCR (quantitative PCR) experiments indicated that MKRN3 did up-regulate the expression of *Pcdh17, Usp32, Itpka, Prkaa2* and *Fam111a*, while down-regulating the expression of *Ripor2, Gnrh1, Ftx, Npepl1* and *Syt2*, in a manner depending on its E3 ligase activity (Supplementary Figure S5B). Note that mRNAs of these DEGs all bore poly(A) tails. Next, we asked whether poly(A) tails of mRNAs were involved in MKRN3-mediated differential expression of these genes. To our knowledge, replication-dependent histone mRNAs, which encode the most abundant histone proteins, are the only known cellular mRNAs that are not polyadenylated, thus lacking poly(A) tails. Indeed, histone mRNAs shows little changes in our RNA-seq data (green colour in Figure 5A), and MKRN3 showed no appreciable effect on the homeostatic levels of *Hist1h1e, Hist1h2ah* and *Hist1h2ad* mRNA in GT1–7 cells (Supplementary Figure S5C), suggesting that poly(A) tails were required in this matter.

Previously, we have demonstrated that MKRN3-mediated ubiquitination of MBD3 disrupted the both the binding of MBD3 to *GNRH1* promoter and also impaired the recruitment of demethylase Tet2 to the specific region, thus promoting DNA methylation, resulting in epigenetic silencing of *GnRH1* (14). Furthermore, cells in each group were treated with Actinomycin D (5 μg/mL) for indicated durations of time (0, 2, 4 or 6 h) to pre-eliminate the effect of MKRN3 on *Gnrh1* transcription. As shown in Figure 5B, the half-life of *Gnrh1* mRNA (*t*_1/2_) in GT1–7 cells that only were transfected with empty vector was found to be 5.79 h, but it dropped to ∼ 3.92 h in GT1–7 cells that ectopically expressed wild-type MKRN3, while in the cells expressing the enzymatically dead MKRN3 mutant (C340G), the *t*_1/2_ was found to be ∼5.70 h. Therefore, it seemed that the E3 Ub ligase activity of MKRN3 was essential for its down-regulated effect on the stability of *Gnrh1* mRNA.

Furthermore, dual luciferase reporter assay was performed with the construct using *mini-CMV* promoter to constitutively express mRNA comprising the ORF from firefly luciferase but flanked by 3′- and 5′-UTR (untranslated region) derived from that of human *GNRH1* mRNA, termed as PGL3-*GNRH1*(UTR) (Figure 5C), while the construct of PRL-TK expressing Renilla luciferase was also introduced as an internal control of mRNA translation. As shown in Figure 5C, reporter assay indicated that the firefly luciferase activity was significantly higher in *MKRN3^−^^/^^−^* HEK293T cells than that of wild-type cells, suggesting a possible suppressive effect of endogenous MKRN3; however, this could be efficiently reversed by re-introduction of wild-type MKRN3 but not E3 ligase dead mutant MKRN3 (C340G) into *MKRN3^−^^/^^−^* HEK293T. Then the mRNA half-lives of the reporter mRNAs were also assessed upon Actinomycin D treatment, the *t*_1/2_ of the reporter mRNAs in *MKRN3^−^^/^^−^* cells transfected with empty vectors or MKRN3 (C340G) were ∼50% longer than that in wild-type cells or in *MKRN3^−^^/^^−^* cells that transfected with MKRN3 (Supplementary Figure S5D). As shown in Figure 5D and Supplementary Figure S5E, PABPC1 up-regulated the expression of *GNRH1(UTR)* luciferase though stabilizing its mRNA, and such an effect could be almost completely abolished by the re-introduced wild-type MKRN3, but not the CPP-associated mutant (C340G). On contrast, PABPC1_4KR_, the PABPC1 mutant defective for MKRN3-mediated ubiquitination, did activate the *GNRH1(UTR)* luciferase activity as wild-type PABPC1 seemingly through stabilizing their mRNAs; however, unlike wild-type PABPC1, the luciferase activity activated by PABPC_4KR_ were no longer susceptible to MKRN3-mediated repression (Figure 5E and Supplementary Figure S5F). Consistent with those mRNAs stability tests, these results clearly indicated that MKRN3-conjugated poly-Ub chains particularly those on the four Lys sites (312, 512, 620 and 625) of PABPC1 were primarily responsible for MKRN3-mediated suppressing effect on the translation of *GNRH1*(UTR) mRNA. Similar results were also observed with the MKRN3-interacting PABPC3 or PABPC4 (Supplementary Figure S5G and H).

The effect of MKRN3-mediated PABPC1 ubiquitination also hold true with the homeostasis of other mRNAs. For example, *Ripor2* and *Npepl1*, the two hits whose homeostatic levels were found to be down-regulated by MKRN3 as revealed by RNA-seq (Figure 5A). As shown in Supplementary Figure S5I, PABPC1 increased the half-lives of the mRNAs, which was reversed by wild-type MKRN3, but not MKRN3 (C340G). Meanwhile, MKRN3 or PABPC1 had little effect on the stability of *Hist1h1e* and *Hist1h2ah* mRNAs, which lacked the poly(A) tails (Supplementary Figure S5J). Therefore, the mRNA poly(A) tails, which were bound by PABPCs, was critically required for the effect of MKRN3 on mRNAs stability and translation.

Primary neurons were also dissected from hypothalamic tissues of wild-type or *Mkrn3^m+/p^^−^* fetal male mice, cultured and treated with Actinomycin D (5 μg/ml) for the indicated times (0, 2, 4 or 6 h) prior to harvest, and the mRNA levels of *Gnrh1* were detected by qPCR. The half-lives of *Gnrh1* mRNA (*t*_1/2_ ∼ 6.25 hours) in *Mkrn3*^m+/p-^ neurons was approximately 35% longer than that in wild-type (*t*_1/2_ ∼ 4.62 h) (Figure 5F). These data clearly indicated that genetic ablation of *Mkrn3* did significantly prolong the stability of *Gnrh1* mRNA. Finally, the influence of MKRN3-PABPC1 interaction on the stability of *Gnrh1* mRNA, and the levels of GnRH1 proteins were also examined. GT1–7 cells were transfected with empty vector, or vectors expressing MKRN3 or MKRN3 (C340G) only, or co-transfected with vectors encoding PABPC1 or PABPC1_4KR_. As shown in Supplementary Figure S5K, MKRN3-mediated the ubiquitination of PABPC1 negatively regulate the expression of endogenous GnRH1 at both mRNA and protein levels.

### MKRN3 attenuates the binding of PABPs to the poly(A) tails of *GNRH1* mRNA and disrupts the formation of translation initiation complex (TIC)

**Figure 6.**
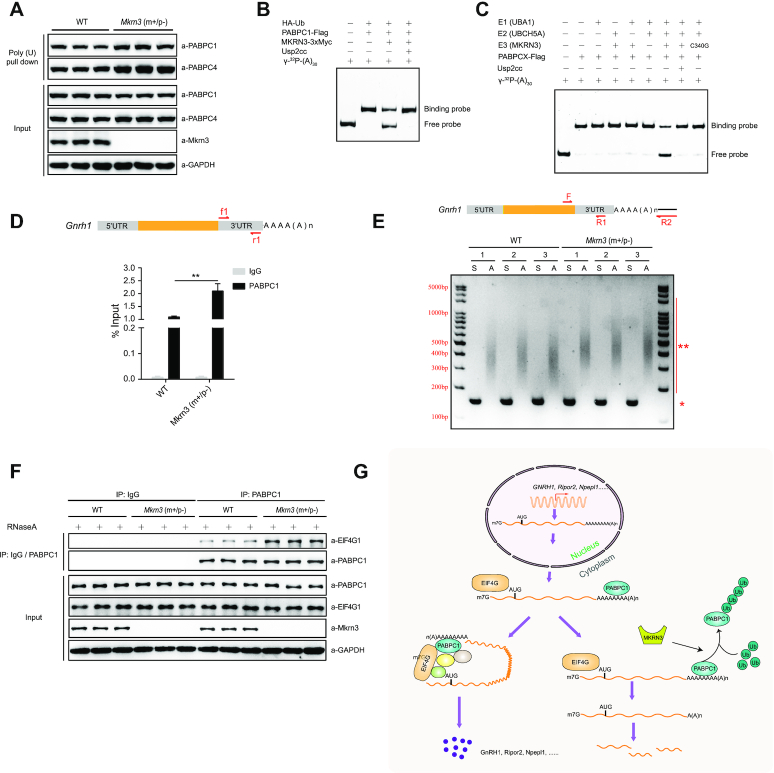
The ubiquitination of PABPs by MKRN3 comprises its binding to the poly(A) of mRNA and the formation of translation initiation complex (TIC). (**A**) More PABPC1 and PABPC4 proteins were recovered with poly(A)-tailed mRNAs in the hypothalamus of *Mkrn3^m+/p-^* mice than those from age-matched wild-type littermates. The total ploy(A)-tailed mRNAs were recovered with poly (U) pull-down assay, in which lysates of hypothalamic tissues were precipitated using poly(U) agarose beads (see *Materials and methods* for details). The poly(A)-bound PABPC1 and PABPC4 were detected by immunoblotting using indicated antibodies, three mice were used in each group. (**B**) MKRN3 mediated the ubiquitination of PABPC1 compromised its binding to (A)_30_ RNA as detected by RNA EMSA. *MKRN3^−^^/^^−^* HEK293 cells were co-transfected with HA-tagged Ub, Flag-tagged PABPC1, and 3xMyc-tagged MKRN3 or not. 24 hours later, cell lysates were immunoprecipitated using anti-Flag affinity gels, treated with Usp2cc or not, and eluted with Flag peptides before subjected to RNA EMSA analysis (see *Materials and methods* for details). Usp2cc, the catalytic core of human deubiquitinase Usp2. (**C**) MKRN3-mediated ubiquitination compromised the affinity of PABPC1 to (A)_30_ RNA. EMSA assay was performed using (A)_30_ RNA probe that labeled with γ-^32^P. Recombinant PABPC1 was subjected to *in vitro* ubiquitination under varying indicated conditions, then immunoprecipitated with anti-Flag affinity gels before subjected to the EMSA assay. (**D**) Ablation of endogenous Mkrn3 promoted the binding of endogenous PABPC1 to *Gnrh1* mRNA in the hypothalamus of *Mkrn3^m+/p^^−^* mice compared to that of wild-type ones. RNA immunoprecipitation assay was done with the hypothalamus of wild-type and *Mkrn3^m+/p^^−^* male mice at post-natal day 15, and PABPC1-bound mRNAs were enriched with anti-PABPC1 antibodies followed by qPCR assays using primers f1+r1. Data were presented as Mean ± SD, two-tailed unpaired *t*-test, *n* = 5. **P < 0.01, very significant difference. (**E**) The poly(A) tail-length of *Gnrh1* mRNAs in the hypothalamus of *Mkrn3^m+/p^^−^* mice were longer than those in wild-type. Total RNAs of hypothalamic tissues were prepared from male mice at post-natal day 15, followed by addition of a limited number of guanosine and inosine residues to the 3′-ends of poly(A)-containing RNAs, to allow reverse transcription-PCR (RT-PCR) amplification using primer R2 (see *Materials and methods* for details). S, gene-specific amplicons to tell the abundance of *Gnrh1* mRNA, using primers F+R1; A, poly(A)-tail-containing amplicons to tell the poly(A) tail-length of *Gnrh1* mRNA, using primers F+R2.*, gene-specific bands; **, poly(A) tail-length bands, three samples in each group. (**F**) Ablation of endogenous Mkrn3 promoted PABPC1-EIF4G1 interaction in the hypothalamus of mice. Co-immunoprecipitation assay were performed with the hypothalamus of wild-type and *Mkrn3^m+/p^^−^* mice at post-natal day 15 using anti-IgG or anti-PABPC1 antibody, treated with RNaseA (50 μg/ml) or not, followed by immunoblotting with indicated antibodies, three samples in each group. (**G**) A model depicting how the MKRN3-PABPC1 axis controls the post-transcriptional switch of mammalian puberty initiation through regulating the stability and translation of mRNAs (including *GNRH1*) in hypothalamic cells.

To assess the potential effect of MKRN3-mediated ubiquitination of PABPs on their binding to poly(A) tail of mRNAs, poly(U)-pull down assay were performed to enrich poly(A)-containing mRNAs as well as its associated proteins. The assay was performed with lysates from the hypothalamus of either wild-type or *Mkrn3^m+/p-^*male mice to examine the effect of endogenous MKRN3 on the binding of PABPs to mRNA poly(A) tails. The specificities of antibodies against PABPC1 or PABPC4 were detected by immunoblotting analysis using recombinant PABPC1-His6 or PABPC4-His6 proteins (Supplementary Figure S6A), and clearly the same amount of poly(U) beads recovered more endogenous PABPC1 or PABPC4 proteins from the hypothalamus tissue lysates of *Mkrn3^m+/p–^* mice than those of wild-type controls (Figure 6A). Such assay was also performed with hypothalamus-derived mouse GT1–7 cells transfected with empty vector or the vector encoding MKRN3 or MKRN3(C340G), and less endogenous PABPC1 or PABPC4 proteins were recovered from the cells transfected with wild-type MKRN3, compared to those of cells transfected with either empty vectors or the E3 ligase dead mutant MKRN3(C340G) (Supplementary Figure S6B).

Meanwhile, an *in vitro* RNA EMSA assay was performed to directly assess the effect of MKRN3-mediated ubiquitination of PABPC1 or PABPC4 on their direct binding to γ-^32^P labeled synthetic (A)_30_ RNA oligomers. To this end, the specificity of the recombinant PABPC1 or PABPC4 towards γ-^32^P labeled (A)_30_ RNA oligos were first checked through assaying their competitive binding to the hot (labeled) and the cold (unlabeled) RNA oligomers of the same sequence (added at an 1:10 ratio, see Supplementary Figure S6C and D). Ubiquitinated endogenous PABPC1 or PABPC4 proteins were first enriched from HEK29T cells, with or without Usp2cc treatment to remove the ploy-Ub chains that they bore, and then subjected to EMSA assay with γ-^32^P labeled (A)_30_ RNA. As shown in Figure 6B and Supplementary Figure S6E, ubiquitinated PABPC1 or PABPC4 manifested much weaker binding to the γ-^32^P labeled (A)_30_ oligos, when compared to that by the respective unmodified PABP proteins. Recombinant PABPC1 or PABPC4 proteins were also subjected to *in vitro* ubiquitination reaction, recovered with anti-Flag affinity gels, and followed by the same EMSA analysis indicated above. The results again demonstrated that ubiquitinated PABPC1 or PABPC4 showed much less affinity to γ-^32^P labeled (A)_30_ RNA as compared to unmodified proteins (Figure 6C and Supplementary Figure S6F). Collectively, these data established that MKRN3-mediated ubiquitination could negatively regulate the bindings of the PABP proteins to the poly(A) tails of mRNAs.

With the particular interest in the homeostasis of *GNRH1* mRNA, we next asked whether and how MRKN3-mediated the ubiquitination of the PABPs would affect their binding to *GNRH1* mRNA, or the poly(A) tail-length of *GNRH1* mRNA. As shown in Figure 6D, RNA immunoprecipitation (RNAIP) assay indicated that the same amounts of anti-PABPC1 did pull down ∼100% more *Gnrh1* mRNAs in MKRN3-deficient (*Mkrn3^m+/p^^−^*) hypothalamus than those from the wild-type controls, suggesting more endogenous PABPC1 proteins were binding to *Gnrh1* mRNA in *Mkrn3^m+/p-^*mice in which MRKN3 was genetically ablated. Similar results were also observed in GT1–7 cells that transfected with empty vector, or the vector encoding MKRN3 or MKRN3(C340G) (Supplementary Figure S6G). To further explore how the MKRN3-PABPs axis might have an effect on poly(A) tail-length of *Gnrh1* mRNA, Poly(A) tail-length assay were performed. PCR amplifications were performed with the reverse-transcribed *Gnrh1* mRNA as templates using the primers F and R1 to specifically amplify the non-poly(A) 3′-UTR regions as internal control, while using primers F and R2 to amplify the region that contains both the specific 3′-UTR region of *Gnrh1* mRNA and its poly (A) tails. As shown in Figure 6E, the poly(A) tail-length of *Gnrh1* mRNA in the hypothalamus of MKRN3-deficient (*Mkrn3^m+/p^^−^*) was longer (amplicons of averagely ∼ 500 bp long) than those of wild-type ones (amplicons of averagely ∼ 400 bp long).

Altogether, Mkrn3-mediated ubiquitination attenuated the binding of PABPs to *Gnrh1* mRNA in the hypothalamus of mice, which also led to the shortened ploy(A) tail-length of these mRNAs.

PABPC1 and EIF4G1 are direct interacting components in the translation initiation complex (TIC) which regulates the overall mRNA translation efficiency, and they are essential for TIC formation (42,43). Then we asked whether MKRN3-mediated ubiquitination affects the interaction between PABPC1 and EIF4G1. Lysates of the hypothalamus from wild-type or *Mkrn3^m+/p^^−^* mice were prepared, and subjected to co-IP assays using anti-IgG or anti-PABPC1 antibodies with or without RNaseA treatment, followed by immunoblotting analysis using specific antibodies to detect the relative abundances of indicated proteins. As shown in Figure 6F, the same amounts of anti-PABPC1 antibodies enriched almost equal amounts of endogenous PABPC1 proteins in both groups, but much more EIF4G1 were pulled down from the *Mkrn3^m+/p^^−^* mice than that of wide-type ones. These data suggested that the ablation of Mkrn3 promoted the formation of PABPC1-EIF4G1 complex.

Altogether, it is thus clear that the ubiquitination of PABPC1 or PABPC4 by MKRN3 negatively regulates the formation of translation initiation complex (TIC), attenuates their binding to poly(A)-tail contained mRNAs, and leads to the shortened poly(A) tail-length of *GNRH1* mRNA. This could collectively contributes to the higher homeostatic levels of *GNRH1* mRNA and protein, when MKRN3 was mutated in CPP patients, ablated in mice or silenced upon puberty initiation (Figure 6G).

## DISCUSSION

Hypothalamic production and secretion of GnRH is believed to mark the switch-on of mammalian puberty, and GnRH is recognized and confirmed to be a master regulator of puberty through activating hypothalamic-pituitary-gonadal (HPG) axis (8,44,45). The expression of GnRH is known to be subjected to multiple factors, including the most recently found three microRNAs, miR-200, miR-155and miR-30, each functioning through distinct mechanisms (8,44). Recently, MKRN3 has emerged as a pubertal repressor, as mutations in *MKRN3* were found in human patient of central precocious puberty (CPP) MKRN3 across different races (2). These findings strongly suggested that the altered expression of Mkrn3 and GnRH in the GnRH neurons was sufficient to modulate the activity of HPG and significantly impact puberty initiation at organism level. Indeed, a strain of Mkrn3 knockout mice were generated in our lab did manifest many phenotypes similar to those symptoms observed in human CPP patients, e.g. increased production of GnRH1, early onset of puberty initiation in both male and females, *etc* (14). Furthermore, through yeast-based genetic screening, we have identified MBD3, an epigenetic reader, as a *bona fide* substrate for MKRN3 and found that MKRN3 conjugates poly-Ub chains onto MBD3 on multiple sites. The ubiquitination of MBD3 by MKRN3 disrupts the interaction between MBD3 and the DNA demethylase Tet2, as well as the binding of MBD3 to *GNRH1* promoter, thus epigenetically silencing the transcription of *GNRH1* and repressing puberty initiation (14). GnRH1 expression was significantly increased when Mkrn3 was deficient in mice, hypothalamus-derived GT-7 neurons, which could be reversed by re-introduction of wild-type MKRN3 but not the CPP-associated mutants (14). These observations have attracted current notion to that epigenetic mechanisms could play critical roles in regulation of mammalian puberty (3,46). Therefore, MKRN3, a regulatory factor of *GNRH1* gene transcription, in GnRH neurons seemed to regulate mammalian puberty initiation through ‘autonomous’ mode of action, without necessarily affecting GnRH1 secretion.

In this study, three Poly(A)-binding proteins (PABPs) family members, including PABPC1, PABPC3 and PABPC4, were identified as novel interacting partners and substrates for E3 ligase MKRN3. PABPs are a class of RNA-binding proteins that regulate numerous aspects of eukaryotic mRNA fate, which comprises the nuclear PABPN1, and the cytoplasmic PABPC1, PABPC3, PABPC4 and PABPC5 (17,47–49). These PABPs share homologous RNA-binding motifs but also have distinct domain structures, and presumably different functions (Figure 1B). The gene expression profiling revealed a tissue-specific pattern; *Pabpc3* (also known as *pabpc6*) was mainly expressed in mouse tissues of testis, spleen and lung, while *Pabpc1* and *Pabpc4* were expressed in all detected tissues (Supplementary Figure S1D). As no *Pabpc3* was detected in mouse brain tissues, ubiquitination of PABPC3 by MKRN3 might not directly contribute to the neuroendocrine phenotypes observed in the Mkrn3 knockout mice. We also found that the PABC domain of PABPs is essential for MKRN3 mediated interaction and ubiquitination (Figures 2A–E and 3A). PABPC1 specifically bounds to the poly(A) tails of mRNA, and exhibits multiple functions (17). Poly(A) tails are added to the majority of mRNAs during 3′ end processing stages and regulate translocation of a completely processed mRNA to the cytoplasm, mRNA translation efficiency and mRNA quality control and degradation, since poly(A) tails shortening is one of the key steps initiating decay of the body of mRNAs (50–55). In hypothalamic neurons of Mkrn3^m+/p−^ mice, the binding of PABPC1 or PABPC4 to Poly(A) of mRNA was significantly elevated as compared to that of wild-type ones, with the poly(A) tail-length of *Gnrh1* mRNA also markedly increased (Figure 6A and D). Consistently, loss of Mkrn3 promoted the binding of endogenous PABPC1 to the 3′-UTR of *Gnrh1* mRNA, extending the stability of *Gnrh1* mRNA in neurons, resulting in promoted translation of *GNRH1*(UTR) luciferase (Figures 5B, C, F, 6D, Supplementary Figures S5D and S6G). Mechanistically, Mkrn3-mediated the ubiquitination of PABPC1 appeared to destabilize *Gnrh1* mRNA by reducing PABPC1 binds to the Poly(A) tail of mRNA and shortening the length of mRNA poly(A) tail (Figure 6E). PABPC1 directly interacts with translation initiation factor EIF4G1 to form translation initiation complex at the 5′-end of the mRNA, which primes the recruitment of ribosome and subsequently initiate mRNA translation (17,18,56,57). Indeed, MKRN3-mediated ubiquitination was found to compromise the PABPC1-EIF4G1 complex formation (Figure 6F), which established MKRN3 as an emerging regulator of mRNA homeostasis and translation in eukaryotes.

In this study, non-polyadenylated replication-dependent histone mRNAs were analyzed and found no deregulation of these mRNAs. As shown in Figure 5A, Supplementary Figure S5B and Supplementary Table S7, we also found that some mRNAs (that were also polyadenylated) were not regulated by MKRN3 in the way as was *GNRH1* mRNA, with mechanisms yet to be further studied.

PABPC1, PABPC3 and PABPC4 comprise four RRMs (RNA recognition motifs), RRM 1–4 (18). While RRM 1 and 2 manifest high-affinity poly(A)-binding, PABPC1-PABPC1 interactions mediated by the proline-rich region also contribute to ordered binding to poly(A) (58); however, RRM 3 and 4 bind to poly(A) only with modest affinity, but also bind AU-rich RNA and mediate protein-protein interaction (18,58). In this work, four Lys residues (312, 512, 620 and 625) in PABPC1 were identified as the major sites for MKRN3-mediated ubiquitination. Among them, the K312 site located in the 4^th^ RRM domain in PABPC1, and the other three sites in the C-terminal non-RRM region of PABPC1 (Figure 4A), which were all previously shown to be dispensable for poly(A) binding. Therefore, the poly-Ub chains that MKRN3 conjugates onto PABPC1, predominantly in K27 and K29 linkages, might disrupt PABPC1-poly(A) binding through creating steric hindrance, rather than directly ubiquitinating the poly(A)-binding motifs.

Altogether, by identifying MBD3 (14) and PABPs as physiological substrates for MKRN3, our findings have delineated the mechanisms for how MKRN3-mediated signaling regulates the epigenetic and post-transcriptional switches of puberty initiation in mammals. Further phenotypical characterization of *Mkrn3* knockout mice is ongoing, and it would be interesting to see how the MKRN3-PABPs axis might regulate cellular processes other than GnRH production. Meanwhile, the generation and use of conditional Mkrn3 knockout mice would definitely help us understand the tissue-specific roles of Mkrn3, in addition to a glimpse into the regulatory mechanisms in mammalian puberty initiation. It is reasonable to believe that there are factors other than MKRN3 that could also modulate the functionality of MBD3 or the PABPs and appreciably affect the expression or secretion of GnRH, the mutations or any other deviations that disrupt their homeostasis or functions could also affect the normal puberty initiation of mammals. That being said, it is possible to stratify the factors currently known to be associated with early or delayed puberty initiation and development in humans through examining their individual effects on MKRN3-MBD3 and MKRN3-PABPCs axis. There are a lot to be explored.

## DATA AVAILABILITY

All data needed to evaluate the conclusions in the paper are present in the paper and/or the supplementary materials. The generated data in this study have been deposited in the Gene Expression Omnibus (GEO, Accession No. GSE160467) and PRIDE (Accession No. PXD022346).

## Supplementary Material

gkab155_Supplemental_Files
