# Supplementary material for: MKRN3-mediated ubiquitination of Poly(A)-binding proteins modulates the stability and translation of GNRH1 mRNA in mammalian puberty
Source: Nucleic Acids Res. 2021 Mar 21;49(7):3796–813. doi: 10.1093/nar/gkab155 (PMC8053111; doi:10.1093/nar/gkab155)
Supplement: gkab155_Supplemental_Files [file gkab155_supplemental_files.zip › Table S1.docx]

**Table S1.** Plasmids used in this study

| pCDNA3.0-MKRN3-3xFlag | pRK5-HA-UB |
| --- | --- |
| pCDNA3.0-MKRN3-3xMyc | pRK5-HA-UB(K6) |
| pCDNA3.0-MKRN3 (C340G)-3xMyc | pRK5-HA-UB(K11) |
| pCDNA3.0-MKRN3 (R365S)-3xMyc | pRK5-HA-UB(K27) |
| pCDNA3.0-MKRN3 (P417I)-3xMyc | pRK5-HA-UB(K29)) |
| pCDNA3.0-MKRN3 (H420Q)-3xMyc | pRK5-HA-UB(K33) |
| pCDNA3.0-PABPC1-Flag | pRK5-HA-UB(K48) |
| pCDNA3.0-PABN1-Flag | pRK5-HA-UB(K63) |
| pCDNA3.0-PABPC3-Flag | pGEX4T-1-MKRN1 |
| pCDNA3.0-PABPC4-Flag | pGEX4T-1-MKRN2 |
| pCDNA3.0-PABPC5-Flag | pGEX4T-1-MKRN3 |
| pmCherry-C1-MKRN3 | pGEX4T-1-MKRN3(1-125) |
| pEGFP-C1-PABPC1 | pGEX4T-1-MKRN3(126-295) |
| pCDNA3.0-PABPC1(K78R)-Flag | pGEX4T-1-MKRN3(296-380) |
| pCDNA3.0-PABPC1(K157R)-Flag | pGEX4T-1-MKRN3(381-507) |
| pCDNA3.0-PABPC1(K188R)-Flag | pGEX4T-1-MKRN3(△126-295) |
| pCDNA3.0-PABPC1(K213R)-Flag | pET28a-Usp2cc-His6 |
| pCDNA3.0-PABPC1(K312R)-Flag | pET28a-PABPC1-Flag-His6 |
| pCDNA3.0-PABPC1(K512R)-Flag | pET28a-PABPC3-Flag-His6 |
| pCDNA3.0-PABPC1(K620R)-Flag | pET28a-PABPC4-Flag-His6 |
| pCDNA3.0-PABPC1(K625R)-Flag | pET28a-PABPC5-Flag-His6 |
| pCDNA3.0-PABPC1(K312-512R)-Flag | pET28a-PABPC1(1-180)-His6 |
| pCDNA3.0-PABPC1(K312-512-620R)-Flag | pET28a-PABPC1(181-380)-His6 |
| pCDNA3.0-PABPC1(K312-512-620-625R)-Flag | pET28a-PABPC1(381-636)-His6 |
| pGL3-GNRH1(UTR)-Luc | pET28a-mUBA1-His6 |
| pRL-TK | pET28a-UBCH5A-His6 |
| pET28a-PABPC5-PABPC1-His6 | pET28a-UBCH7-His6 |
| pET28a-PABPC1-PABPC5-His6 |  |
