# Supplementary material for: MKRN3-mediated ubiquitination of Poly(A)-binding proteins modulates the stability and translation of GNRH1 mRNA in mammalian puberty
Source: Nucleic Acids Res. 2021 Mar 21;49(7):3796–813. doi: 10.1093/nar/gkab155 (PMC8053111; doi:10.1093/nar/gkab155)
Supplement: gkab155_Supplemental_Files [file gkab155_supplemental_files.zip › Table S2.docx]

**Table S2.** Antibodies used in this study

| **Antibody** | **Source** | **Company** | **Catalog** |
| --- | --- | --- | --- |
| MKRN3 | R | Sigma-Aldrich | HPA029494 |
| PABPC1 | R | Proteintech | 10970-1-AP |
| PABPC4 | R | Proteintech | 14960-1-AP |
| Ubiquitin | M | Santa Cruz | sc-8017 |
| EIF4G1 | R | Abcam | ab2609 |
| His Tag | R | Proteintech | 66005-1-Ig |
| Myc Tag | R | Sigma-Aldrich | SAB4301136 |
| Myc Tag | M | Proteintech | 60003-2-Ig |
| HA tag | R | Sigma-Aldrich | SAB4300603 |
| Flag Tag | R | Proteintech | 20543-1-AP |
| GAPDH | M | Proteintech | [60004-1-Ig](https://www.ptglab.com/products/GAPDH-Antibody-60004-1-Ig.htm) |
| Anti-Flag Affinity Gel | M | Sigma-Aldrich | A4596 |
| Anti-HA Affinity Gel | M | SIGMA | E6779 |
| M，mouse R，Rabbit | | | |
