# Supplementary material for: MKRN3-mediated ubiquitination of Poly(A)-binding proteins modulates the stability and translation of GNRH1 mRNA in mammalian puberty
Source: Nucleic Acids Res. 2021 Mar 21;49(7):3796–813. doi: 10.1093/nar/gkab155 (PMC8053111; doi:10.1093/nar/gkab155)
Supplement: gkab155_Supplemental_Files [file gkab155_supplemental_files.zip › Table S3.docx]

**Table S3.** Primers used for RT-PCR, quantitative PCR (qPCR), Poly(A) tail-length assay or RNAIP-qPCR in this study

| **Primers for RT-PCR (5’-3’)** | |
| --- | --- |
| mGapdh-F | GGCAAGGTCATCCCAGAGCT |
| mGapdh-R | CCAGGAAATGAGCTTGACAAAG |
| mMkrn3-F | CTCCTGGTGACTCCAGGCCC |
| mMkrn3-R | CCGCCACTTCCTGAGTCGGG |
| mPabpc1-F | CAGAGCTTGGAGCCAGGGCA |
| mPabpc1-R | ACAGCTTTCTGCGCATCTTCA |
| mPabpc3-F | GCCCATCCTCTCCATCCGGGTG |
| mPabpc3-R | CAGGAAAGGATGTTACCAAAGGCAGAAA |
| mPabpc4-F | TAGCCCTGCGGGGCCTGTGC |
| mPabpc4-R | ACAAGATGTTTCCAAAGGCAGAGA |
| **Primers for qPCR (5’-3’)** | |
| mGapdh-F | AGAGTGTTTCCTCGTCCCGTAGACA |
| mGapdh-R | CGTTGAATTTGCCGTGAGTGGAGTC |
| mGnrh1-F | GAAAGAGAAACACTGAACACTTGGT |
| mGnrh1-R | TCCTCTTCAATCAGACTTTCCAGAG |
| Firefly-F | GCCGGTGTTGGGCGCGTTATTTATC |
| Firefly-R | CCCCTTTTTGGAAACGAACACCAC |
| Renilla-F | CATGGCCTCGTGAAATCCCGTTAG |
| Renilla-R | CTTGGCACCTTCAACAATAGCATTGG |
| Pcdh17-F | TGAAGGTCCCAACGGAGAAGTC |
| Pcdh17-R | GCTGGGATTGGGTTAGGTCCTA |
| Usp32-F | TGGCTACTGAAGGCCCAATCC |
| Usp32-R | GCAGCAGGCTGATAACCCACA |
| Itpka-F | TCGGAGCCTGAACACTACTGC |
| Itpka-R | CAAGGCCCATCGAAGCCATC |
| Prkaa2-F | GCAAACATGGGCGGGTTGAAG |
| Prkaa2-R | CTTAGCGTTCATCTGGGCATCCA |
| Fam111a-F | GTGGTCAAACATCTTTCGAGGGTCAG |
| Fam111a-R | GGTGGCACAGCCTGCATTTC |
| Ripor2-F | CAACCGCAGCTACAAGGAATACAC |
| Ripor2-R | CACCTCTGCCGACCATACTTCAT |
| Ftx-F | GGAGGCAGAAGAGAGACCAGAG |
| Ftx-R | CATCAGTCTTGAGTTGGGCTCCC |
| Npepl1-F | ACTCTTCACACACCGCTCAGG |
| Npepl1-R | GCTGCTAGTCGGACACCTTCTG |
| Syt2-F | CGAGGAGGAGAAAGAGCCAGAG |
| Syt2-R | CTGGGAGGAGGAAGACTTTGACA |
| Hist1h1e-F | CCTCCGGCTCCTTCAAACTCAAC |
| Hist1h1e -R | GTCTTCTTGGTGCTCTTCTTGGC |
| Hist1h2ah -F | GCCCGCGACAACAAGAAGAC |
| Hist1h2ah -R | CTTGGCCTTGTGGTGGCTCT |
| Hist1h2ad -F | TGGGCCGCGTGCACCGGCTG |
| Hist1h2ad -R | GCTGCAGGTGGCGCGGGATG |
| **Primers for Poly(A) tail-length assay (5’-3’)** | |
| mGnrh1-F | TCGAATGTACTGTCCACTGGCC |
| mGnrh1-R1 | GGGTATAAAAACGCTCAAGCTTACAC |
| **Primers for RNAIP-qPCR (5’-3’)** | |
| mGnrh1-f1 | GCACTGGCCCAGGTGGATCC |
| mGnrh1-r1 | TATGAAATCTACGCTGCTGGGTA |
