# Supplementary figures and images for: MKRN3-mediated ubiquitination of Poly(A)-binding proteins modulates the stability and translation of GNRH1 mRNA in mammalian puberty
Source: Nucleic Acids Res. 2021 Mar 21;49(7):3796–813. doi: 10.1093/nar/gkab155 (PMC8053111; doi:10.1093/nar/gkab155)

A

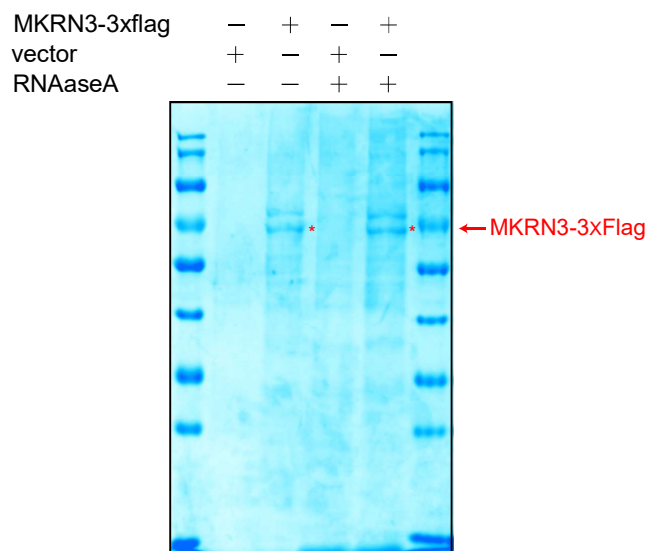

B

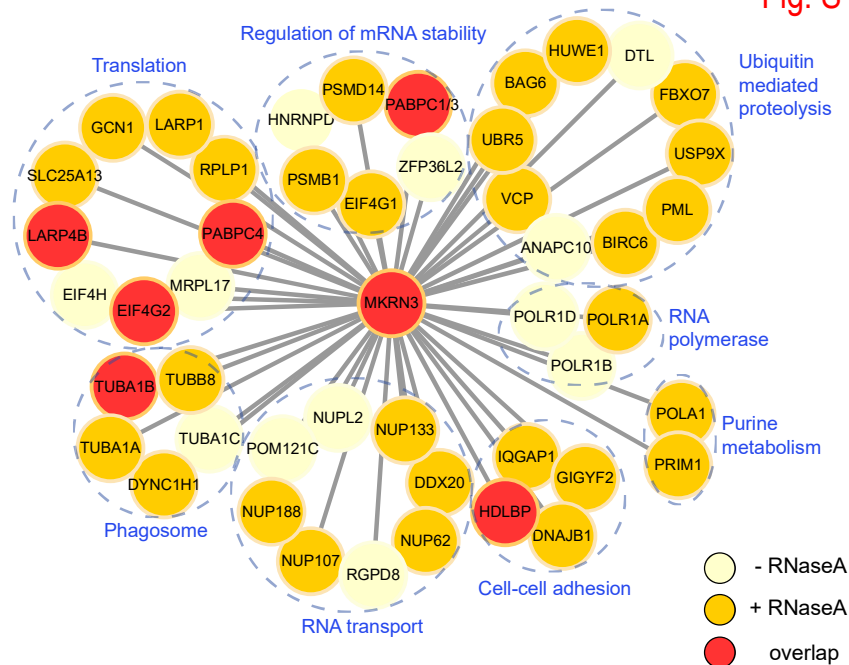

C

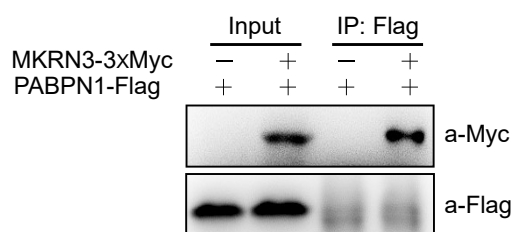

D

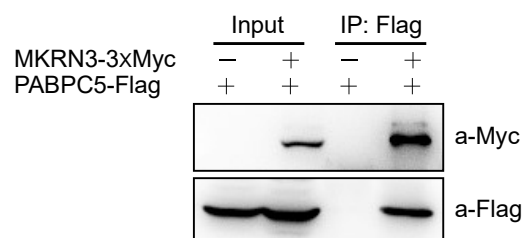

E

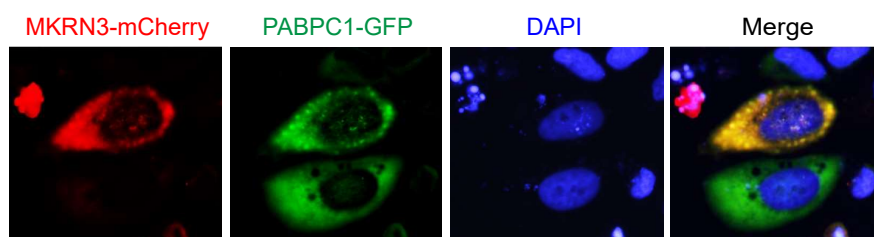

F

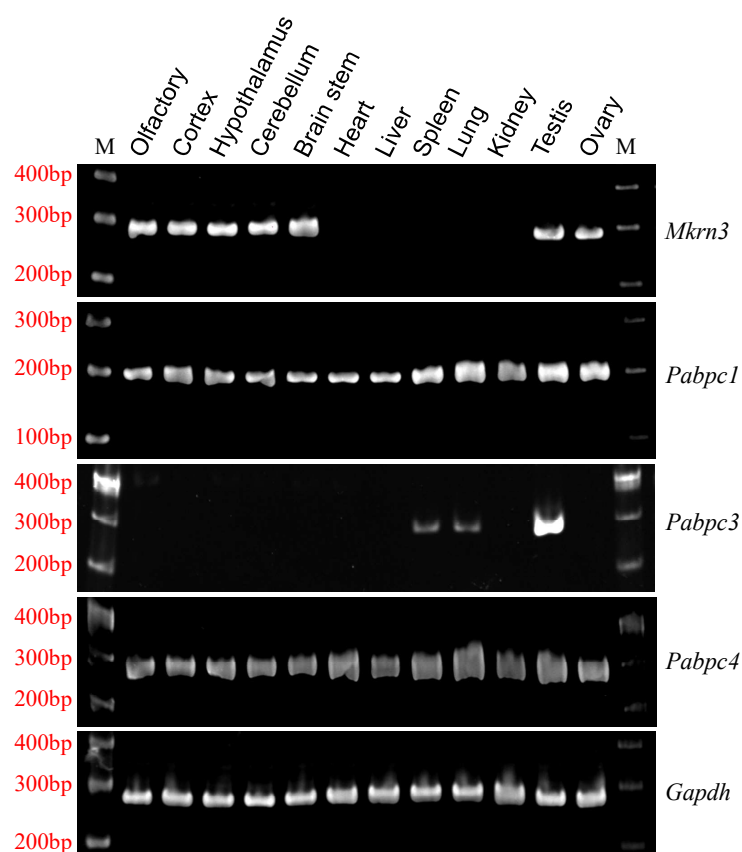

A

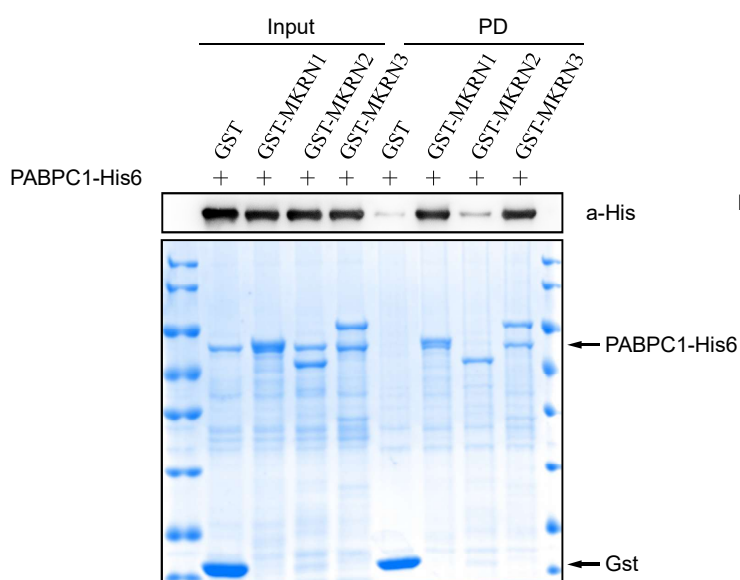

B

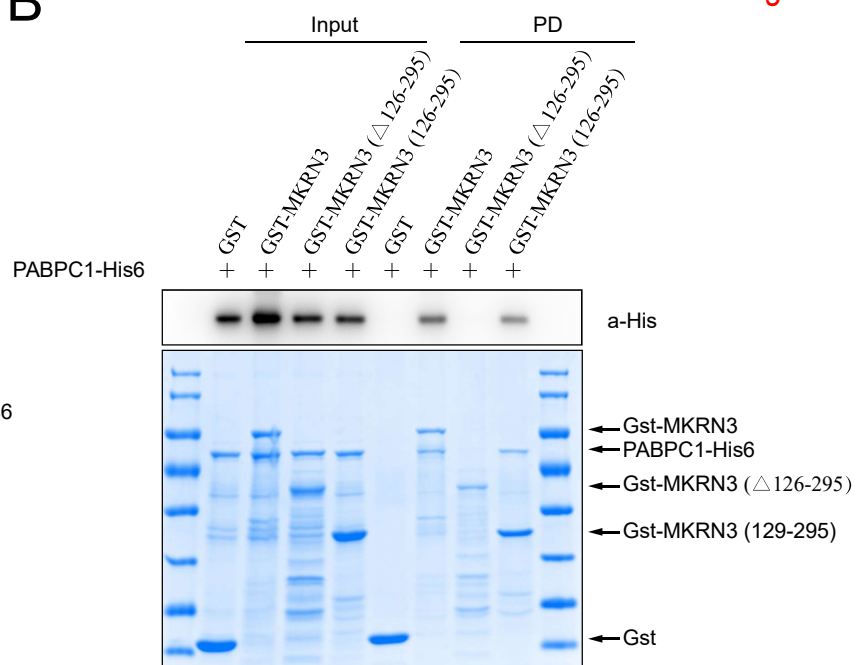

C

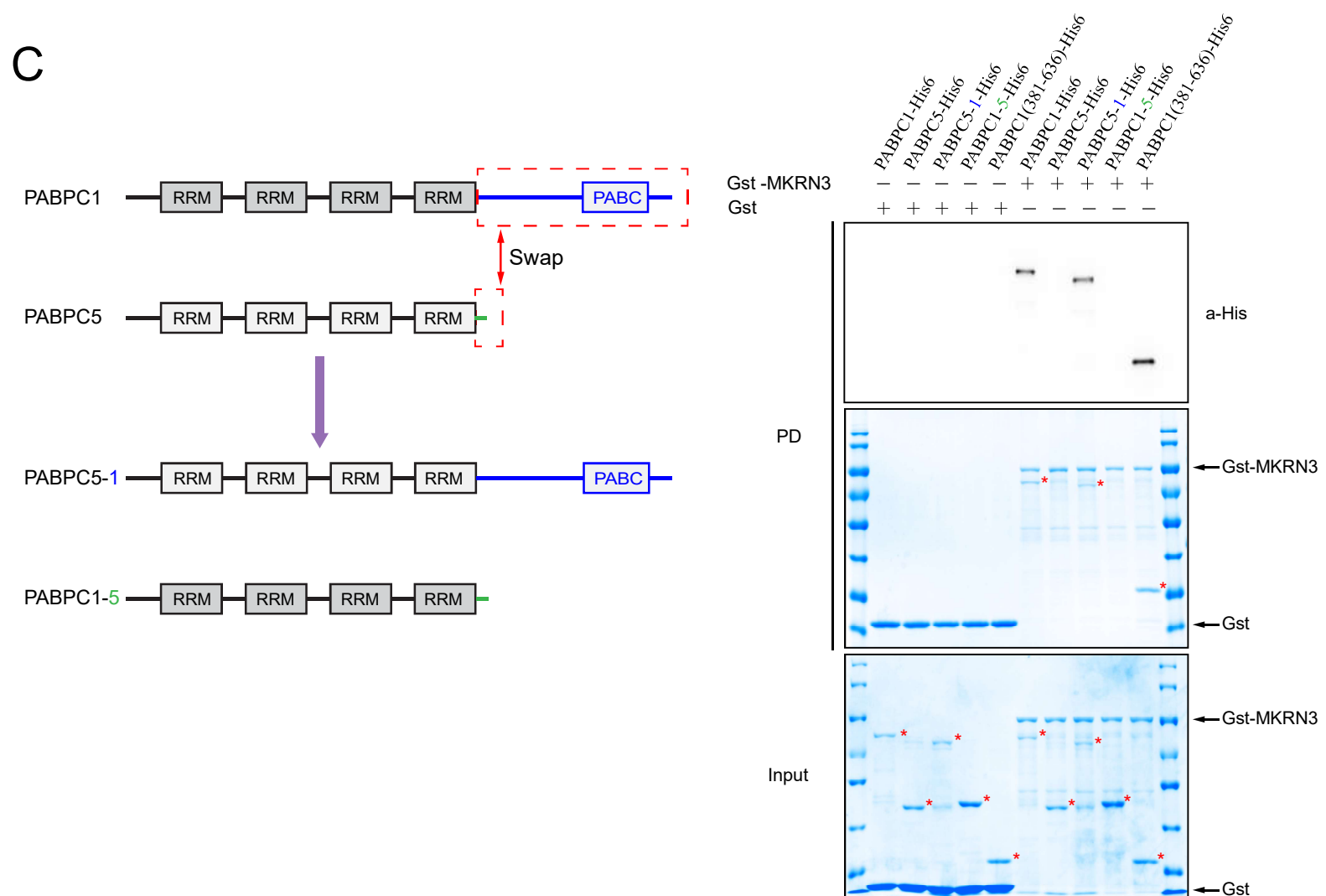

A

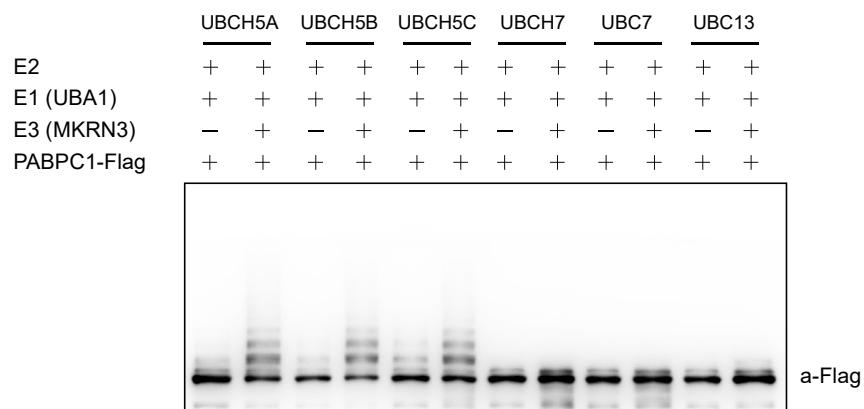

B

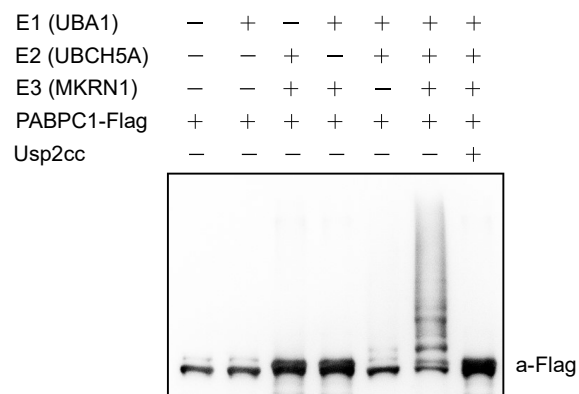

C

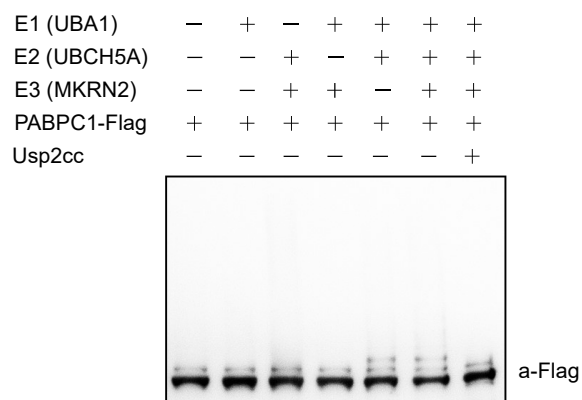

D

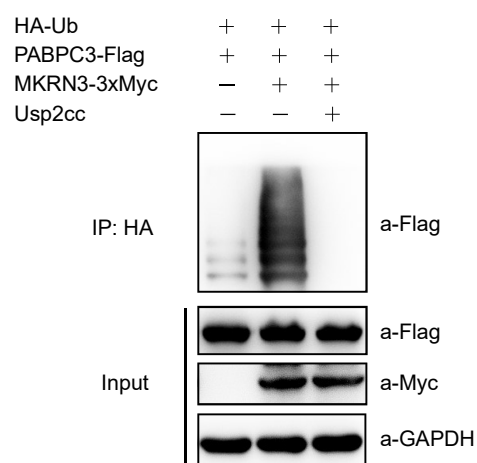

E

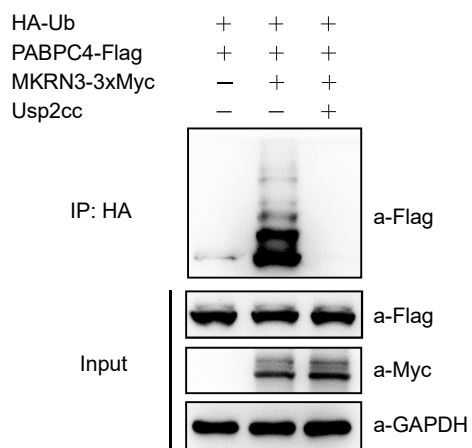

F

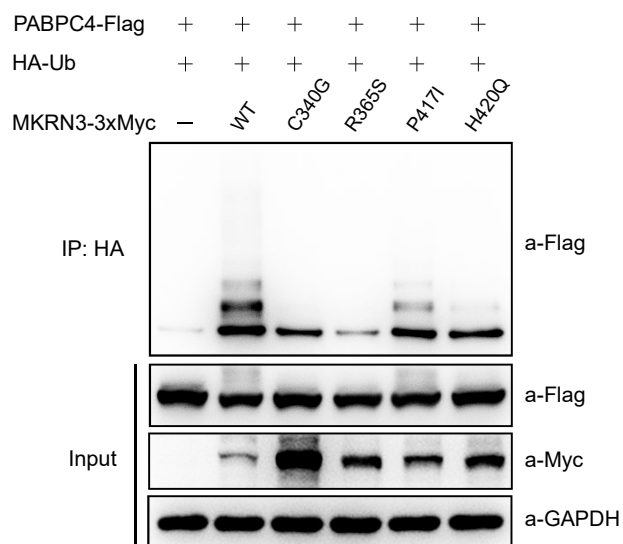

A

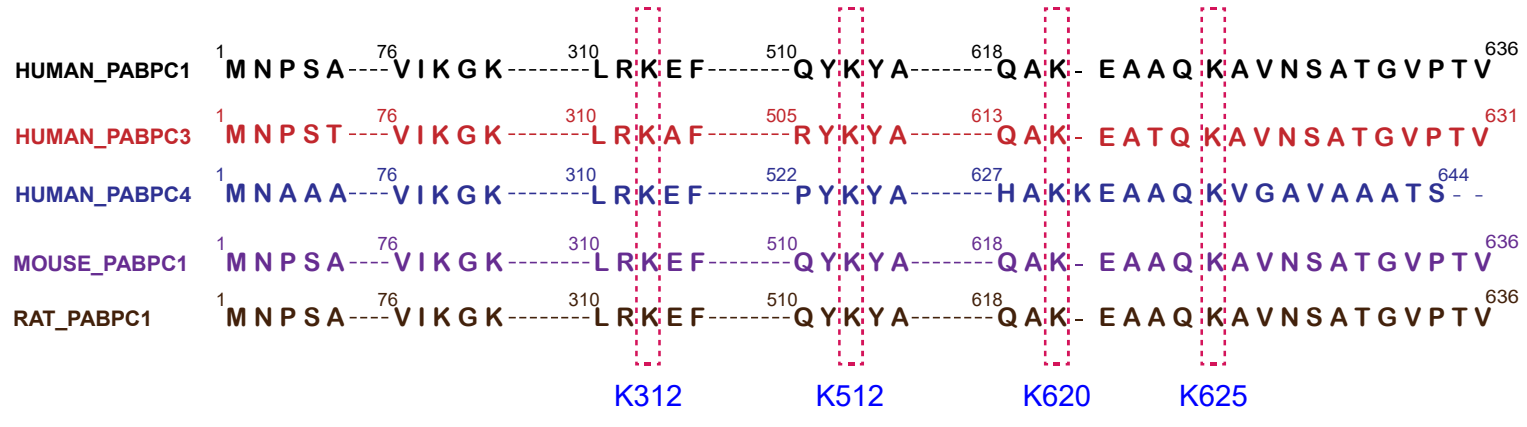

B

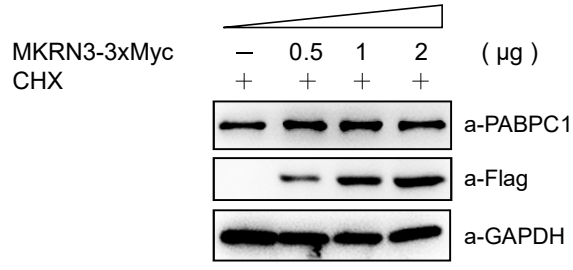

C

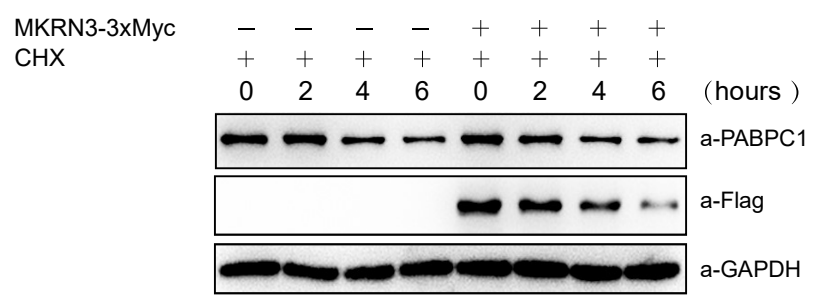

A

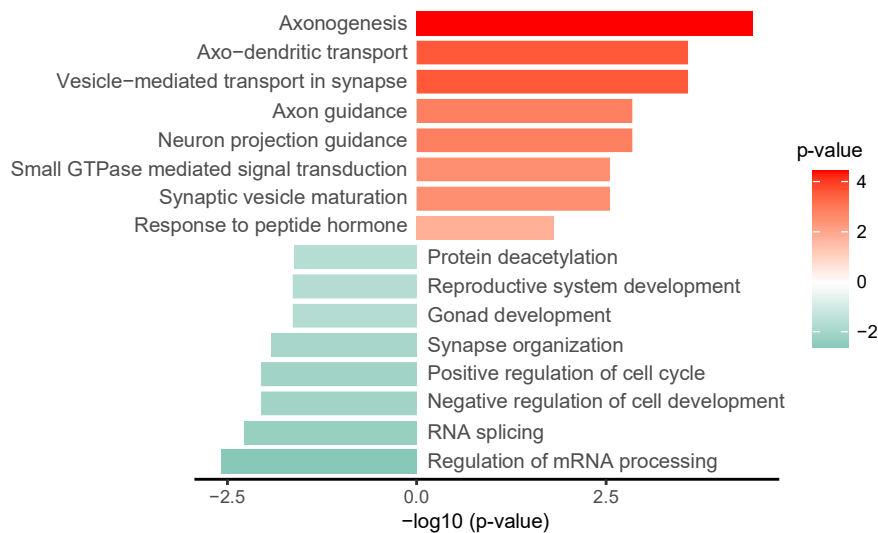

B

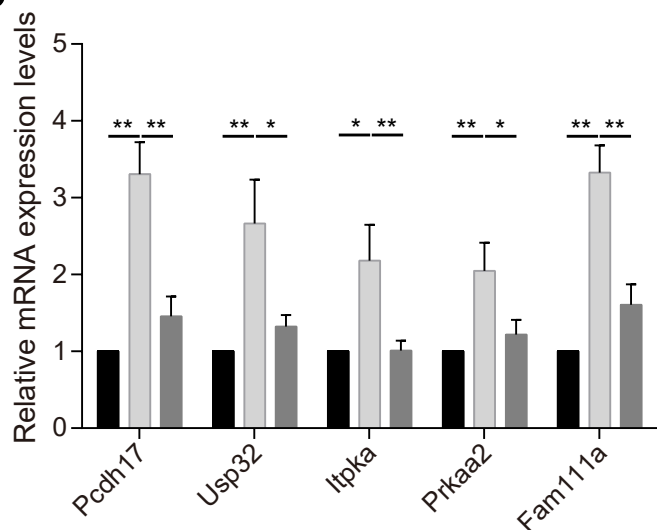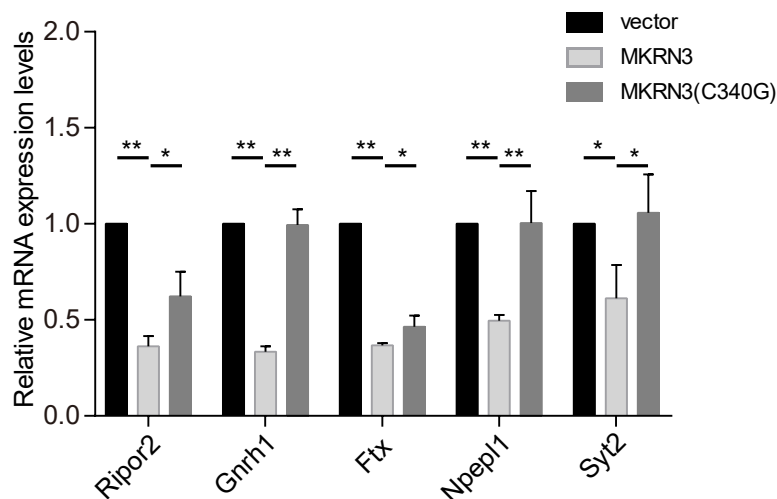

C

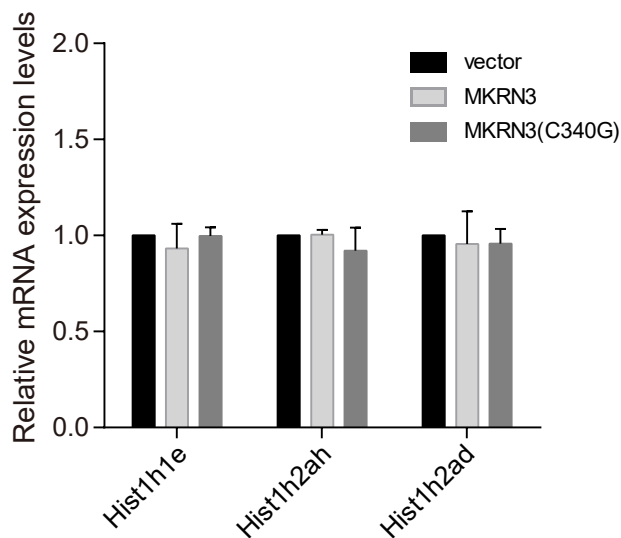

D

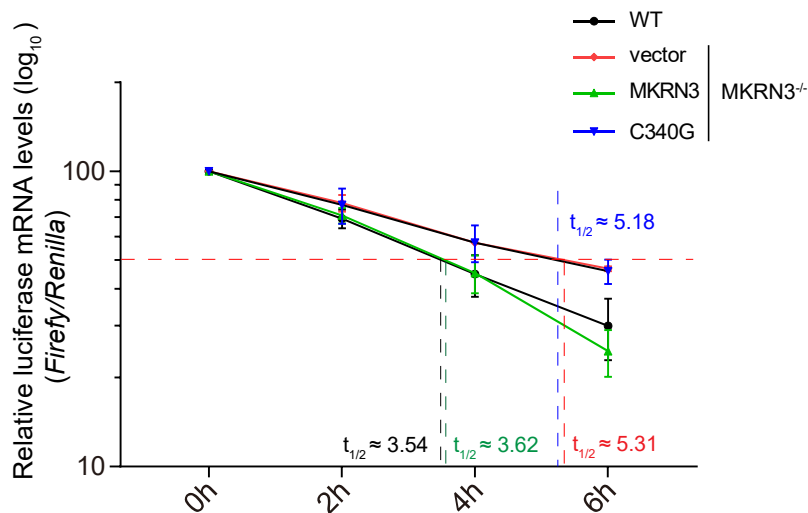

E

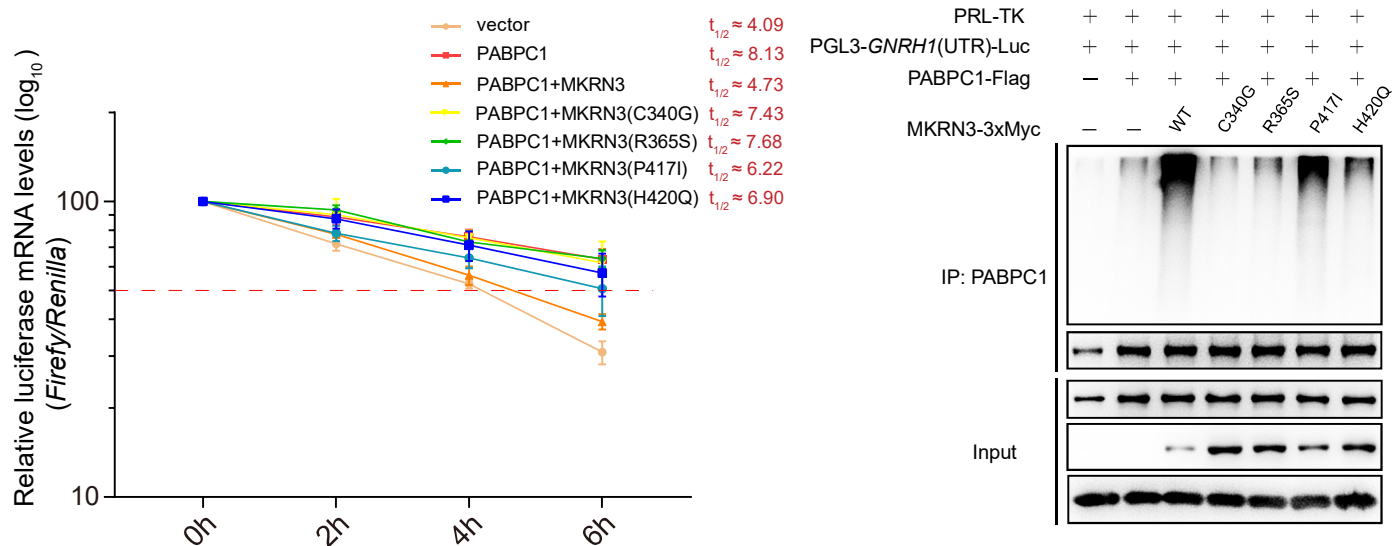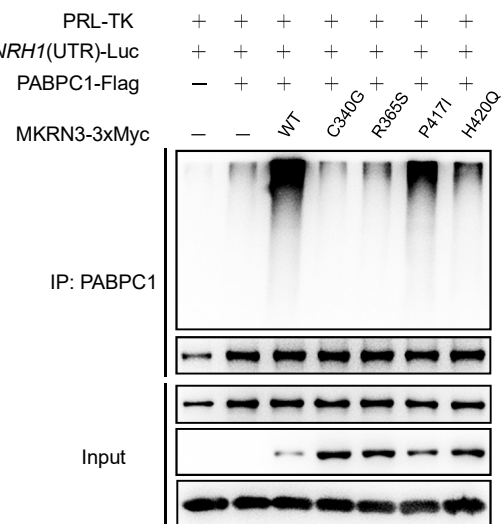

F

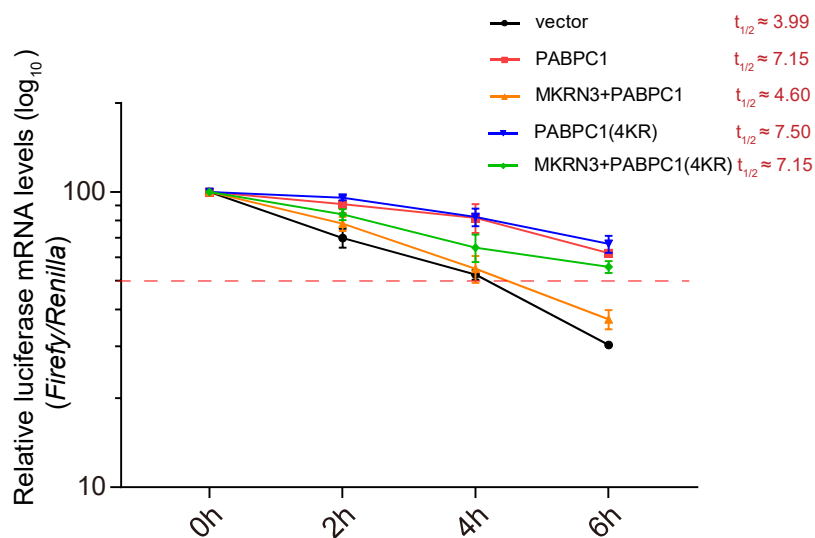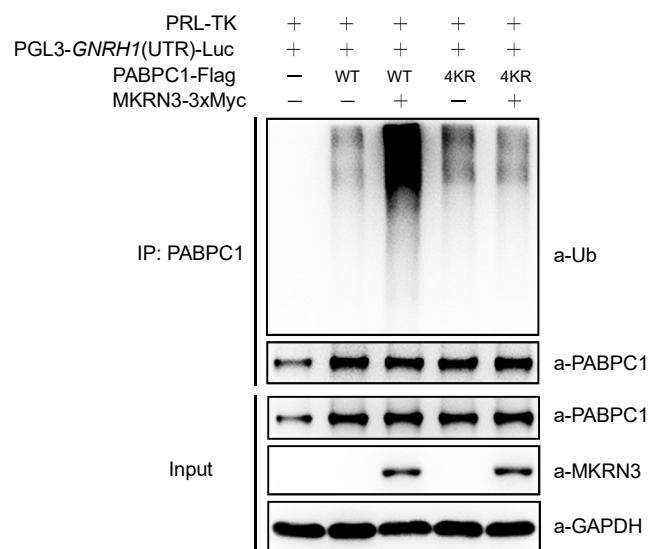

G

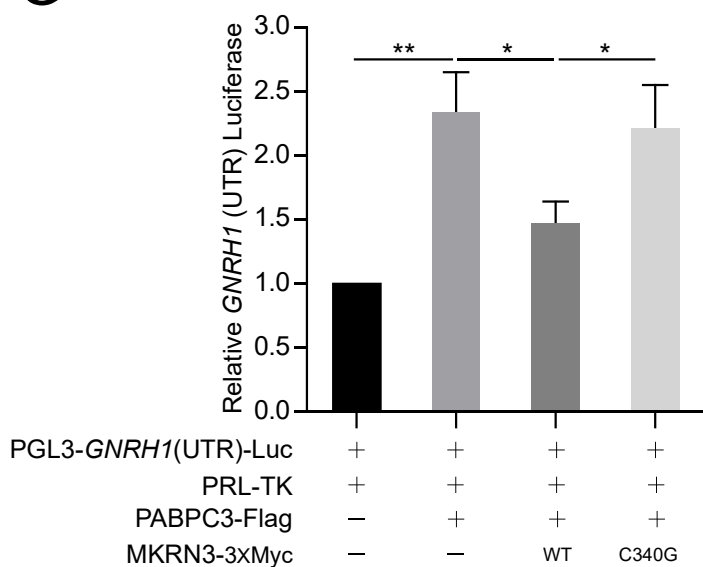

H

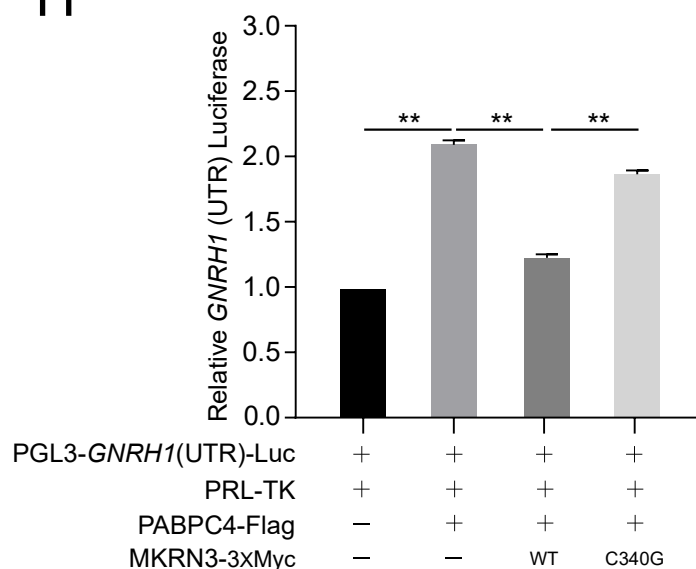

I

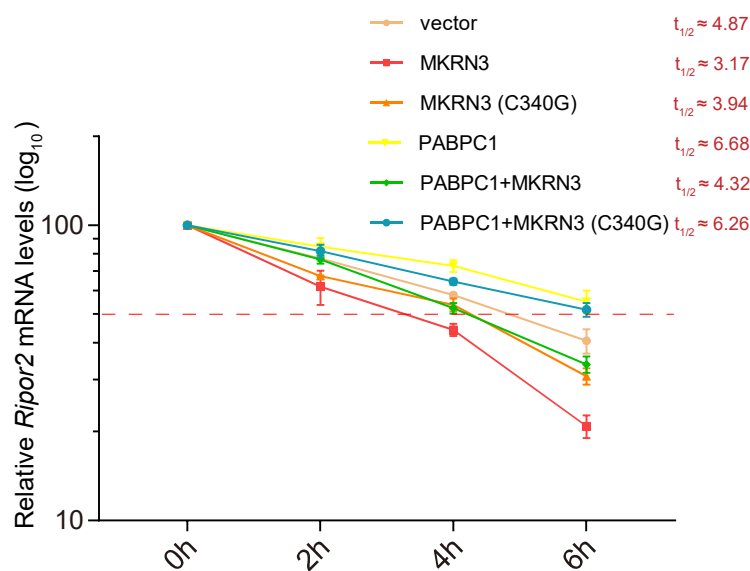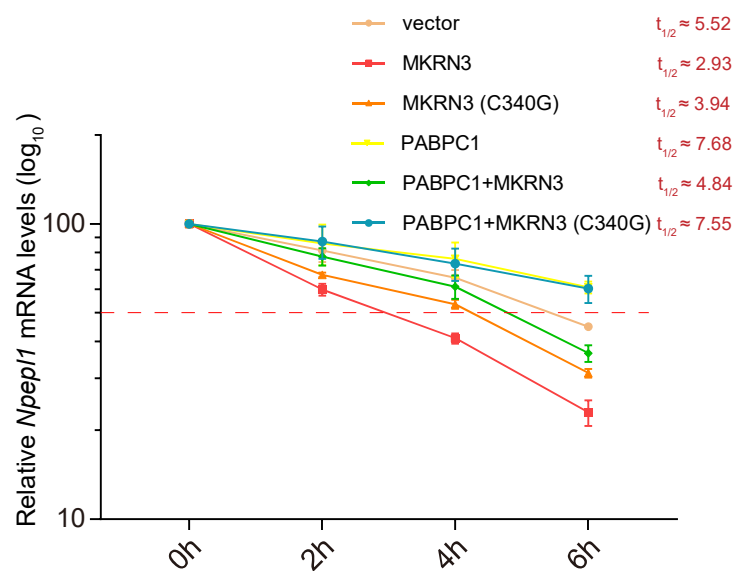

J

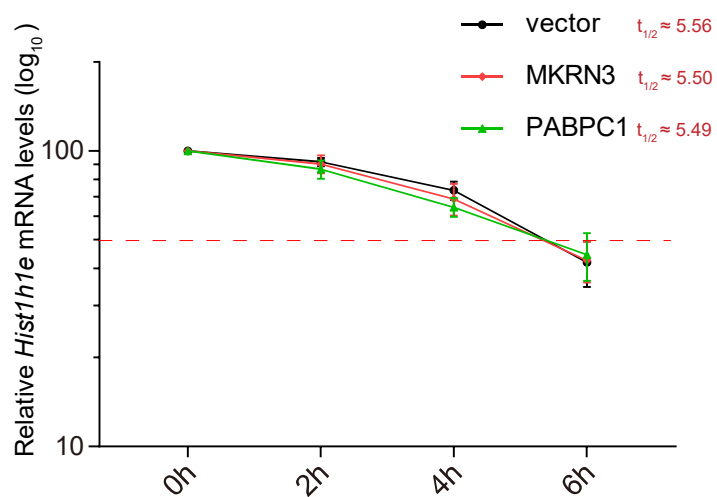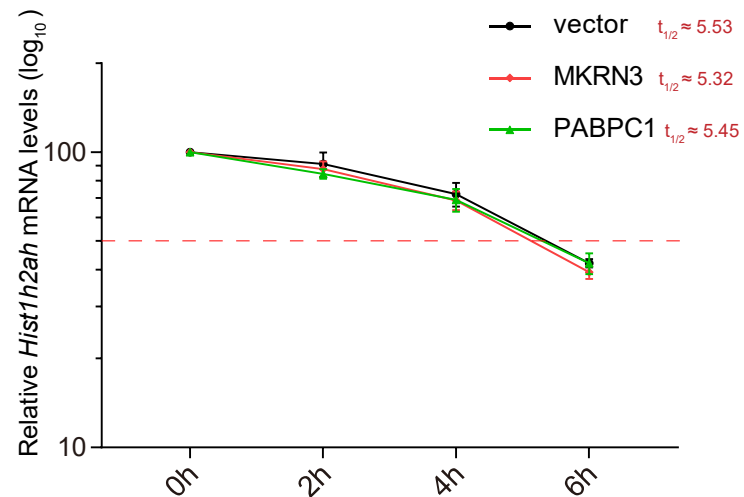

K

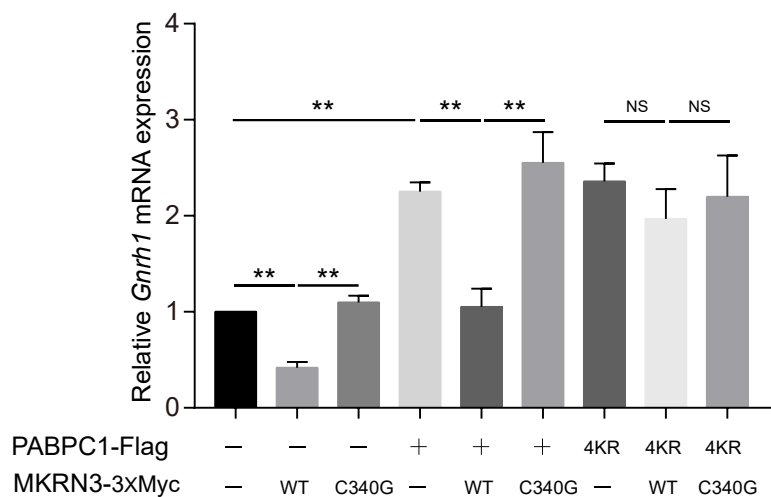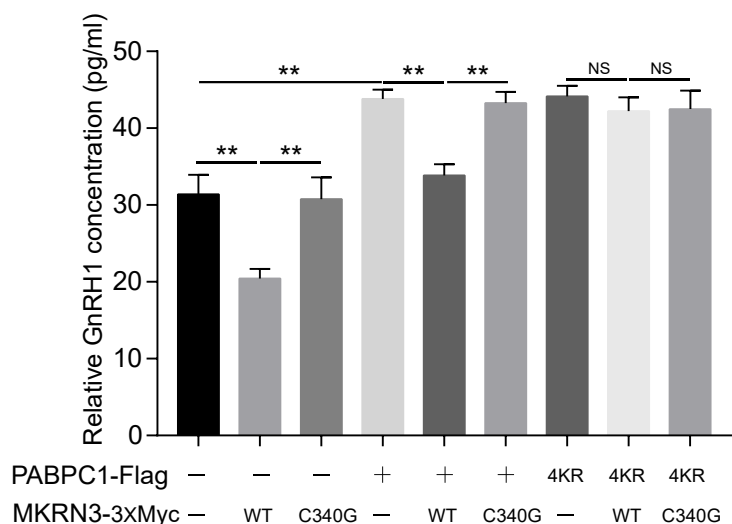

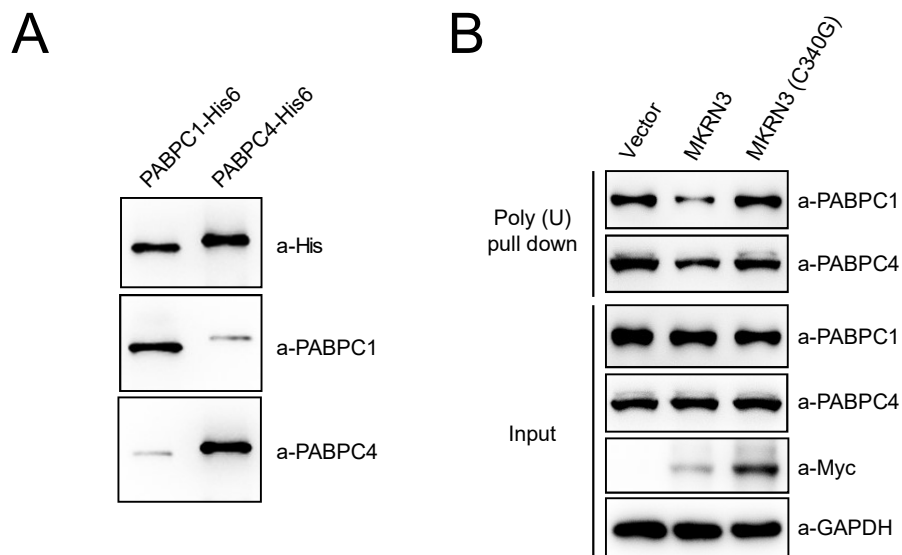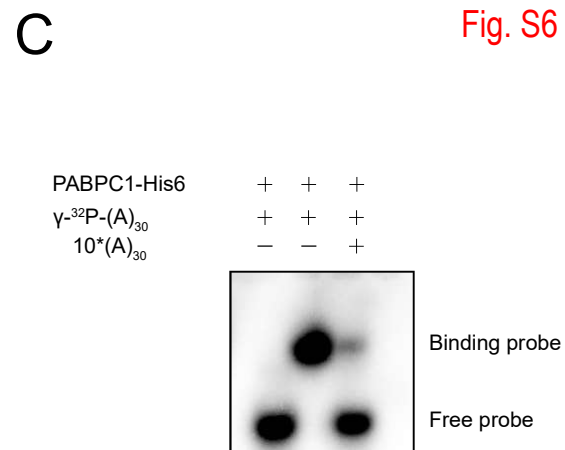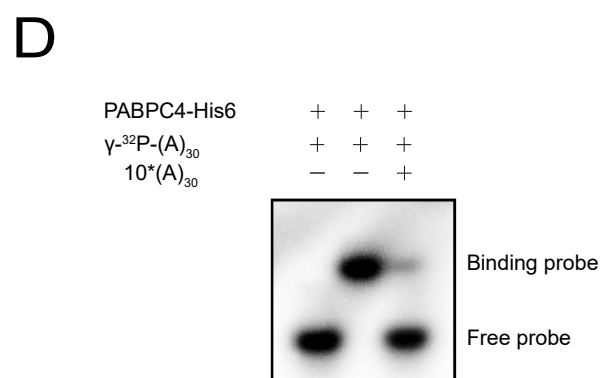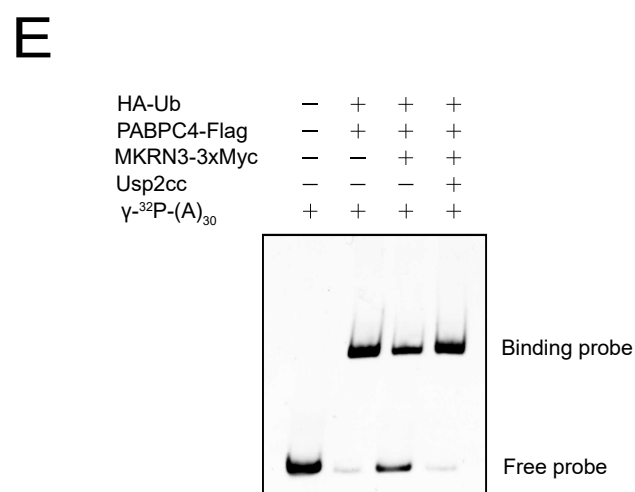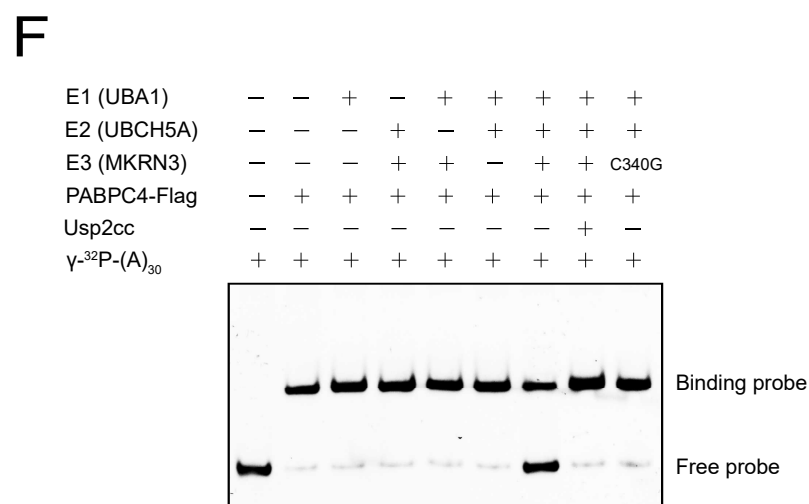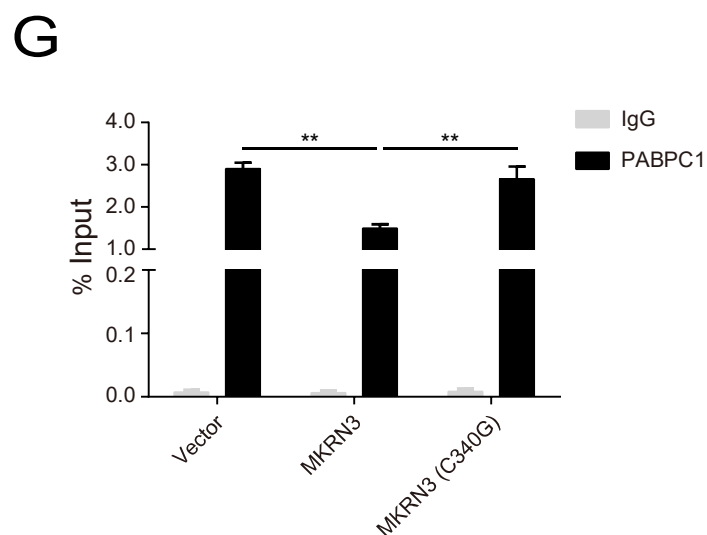

Supplement: gkab155_Supplemental_Files [file gkab155_supplemental_files.zip › supplementary Figure 1-6.pdf]
