# Supplementary material for: MKRN3-mediated ubiquitination of Poly(A)-binding proteins modulates the stability and translation of GNRH1 mRNA in mammalian puberty
Source: Nucleic Acids Res. 2021 Mar 21;49(7):3796–813. doi: 10.1093/nar/gkab155 (PMC8053111; doi:10.1093/nar/gkab155)
Supplement: gkab155_Supplemental_Files [file gkab155_supplemental_files.zip › supplementary figure legends.doc]

**Legends for Supplementary Figures**

Figure S1 to Figure 1

Figure S2 to Figure 2

Figure S3 to Figure 3

Figure S4 to Figure 4

Figure S5 to Figure 5

Figure S6 to Figure 6

**Figure S1. MKRN3 forms complex with poly(A)-binding protein (PABP) family members.**

**(A)** The Commassie Blue gel staining of MKRN3-interacting proteins that prepared from co-immunoprecipitation assay. HEK293T cells were ectopically expressing 3XFlag-tagged MKRN3 or Flag tagged empty vectors. 48 hours later, cells lysates treated with or without RNaseA (50μg/ml) were incubated with anti-Flag affinity gels, and the recovered proteins were resolved on SDS-PAGE and visualized with Commassie Blue gel staining.

**(B)** Gene Ontology (GO) analysis indicated the overlapped MKRN3-interacting proteins that identified by mass spectrometry analysis with samples treated with or without RNaseA. Proteins enriched more than eight folds by MKRN3 compare to empty vectors were selected and subjected to GO analysis. The overlapped proteins included PABP family members- PABPC1, PABPC3 and PABPC4.

**(C-D)** Detect the interaction of ectopically expressed MKRN3 with PABPN1 (**C**), or PABPC5 (**D**) in HEK293T cells. Cells transfected with the indicated plasmids were subjected to co-immunoprecipitation assay using anti-Myc antibody, followed by immunoblotting with anti-Myc or anti-Flag antibody.

**(E)** EGFP tagged PABPC1 co-localized with mCherry tagged MKRN3 in Hela cells. Hela cells were co-transfected with plasmids encoding MKRN3-mCherry and PABPC1-EGFP, the nuclear of cells was visualized with DAPI staining, and then the fluorescence were detected by microscope BX51. EGFP, enhanced green fluorescent protein; mCherry, bright red monomeric fluorescent protein.

**(F)** Gene expression profiling analysis of *Mkrn3*, *Pabpc1, Pabpc3* and *Pabpc4* in different tissues from 15-day-old mice, as revealed by reverse transcription PCR (RT-PCR) analysis.

**Figure S2. MKRN3 directly interacts with the PABC domain of PABP family members.**

**(A)** The MAKORIN family members MKRN1 and MKRN3, but not MKRN2, directly interacted with PABPC1. GST pull-down assays were performed with recombinant Gst-MKRN1, MKRN2 or MKRN3, and PABPC1-His6. PD, GST pull-down.

**(B)** The middle region (126-295aa) of MKRN3 is essential for its binding to PABPC1. GST pull-down assays were performed with recombinant Gst-MKRN3 or its fragments, and PABPC1-His6.

**(C)** The C-terminal region of PABPC1 is essential for its binding to MKRN3 as reveled by classical ‘swap’ experiments. The C-terminal region of PABPC1 was exchanged with the C-terminal region of PABPC5, and the recombinant proteins were purified. GST pull-down assays were performed with recombinant Gst-MKRN3 and PABPC1-His6, PABPC1 (381-636)-His6, PABPC5-His6 or the two ‘swap’ His6-tagged proteins. *, His6 tagged proteins.

**Figure S3. MKRN3 ubiquitinates PABP family members that contain PABC domain and CPP-associated mutations of MKRN3 comprised this ubiquitination.**

**(A**) UBCH5 family members including UBCH5A, UBCH5B and UBCH5C supported the ubiquitination of PABPC1 mediated by MKRN3. An *in vitro* ubiquitination assay were performed with recombinant His6-tagged UBCH5A, UBCH5B, UBCH5C, UBCH7, UBC7 or UBC13, together with other components. Ubiquitinated PABPC1 was detected by immunoblotting using anti-Flag antibody.

**(B-C**) MKRN1(**B**), but not MKRN2(**C**), ubiquitinated PABPC1 *in vitro*. An *in vitro* ubiquitination assay was carried out using the recombinant Flag tagged PABPC1, His6-tagged UBA1 (E1) and UBCH5A (E2), and Gst-tagged MKRN1 or MKRN2 (E3), together with the indicated components. Usp2cc, the catalytic core of human deubiquitinase Usp2.

**(D-E)** Ectopically expressed PABPC3 (D) or PABPC4 (E) was ubiquitinated by 3XMyc tagged MKRN3. *MKRN3^-/-^* HEK293T cells were co-transfected with indicated plasmids. 24 hours after transfection, cell lysates were immunoprecipitated with anti-HA affinity gels, followed by immunoblotting using the indicated antibodies.

**(F)** CPP-associated mutations in MKRN3 compromised its E3 Ub ligase activity towards PABPC4. *MKRN3^-/-^* HEK293T cells were co-transfected indicated plasmids. 24 hours after transfection, cell lysates were immunoprecipitated with anti-HA affinity gels, followed by immunoblotting using anti-Flag to detect ubiquitinated PABPC4.

**Figure S4. MKRN3 mediates non-proteolytic ubiquitination of PABPC1 at multiple sites.**

**(A)** Four major (Lys) sites for the ubiquitination of PABPC1 mediated by MKRN3 are conserved in PABP family members that contain PABC domain (PABPC1, PABPC3 and PABPC4) across human, mouse and rat.

**(B-C)** MKRN3 showed no obvious effect on the stability of PABPC1. (**B**) HEK293T cells were ectopically expressed MKRN3 at different dose (0, 0.5, 1.0 and 2.0 µg), treated with 100.0 µg/ml Cycloheximide (CHX) for 4 hours, and then the protein levels of PABPC1 were detected by immunoblotting 24 hours later. (**C**) HEK293T cells were ectopically expressed MKRN3, treated with 100µg/ml CHX during different time (0, 2, 4 or 6 hours), and then the protein levels of PABPC1 were detected by immunoblotting.

**Figure S5. The ubiquitination of PABPs by MKRN3 attenuates the stability of *GNRH1* mRNA**

**(A)** Gene Ontology (GO) enrichment of MKRN3 up-regulated or down-regulated genes identified by RNA-seq. Genes with adjust p-value ≤0.05 and fold changes ≥2 were considered as differentially expressed genes (DEGs) and involved in pathway enrichment analysis.

**(B-C)** Detection the expression levels of top five MKRN3 up-regulated and down-regulated genes (**B**), and histone genes (**C**) by quantitative PCR (qPCR). GT1-7 cells were transfected with empty vector, or vector expressing MKRN3 or MKRN3 (C340G). 48 hours later, total mRNAs were extracted and subjected to quantitative PCR (qPCR) analysis. The indicated gene signals were normalized to that of *Gapdh*. Data were presented as Mean ± SD, one-way ANOVA with Bonferroni post-hoc test. **P* < 0.05, significant difference; ***P* < 0.01, very significant difference, three independent experiments.

**(D)** MKRN3 destabilized the mRNA of human *GNRH1* (UTR) luciferase reporter. Human *GNRH1* (UTR) luciferase reporter, as well as internal control reporter (PRL-TK), were transfected into wild-type or MKRN3^-/-^ HEK293T cells that ectopically expressing empty vector, MKRN3 or MKRN3 (C340G), and treated with Actinomycin D (5 µg/mL) to block de novo transcription of mRNAs at different time points (0, 2h, 4h or 6h) after 36 hours transfection. Total mRNAs were extracted and subjected to real-time qPCR with *Firefly* gene signals normalized to that of *Renilla*. Each sample was normalized to 100% at time zero, and the half-lives of *Firefly* mRNA (t_1/2_) were calculated. Data were presented as Mean ± SD, three independent experiments.

**(E)** CPP-associated MKRN3 mutations attenuates the destabilized effect on human *GNRH1* (UTR) luciferase reporter mRNA. *MKRN3^-/-^* HEK293T cells were co-transfected with plasmids encoding *GNRH1* (UTR) luciferase, PRL-TK, PABPC1, wild-type MKRN3 or the indicated mutants, and treated with Actinomycin D at indicated time points. Total mRNAs were extracted, qPCR were done, and the half-lives of *Firefly* mRNA (t_1/2_) were calculated. The ubiquitination statuses of PABPC1 were also detected by immunoblotting in each group. Data were presented as Mean ± SD, three independent experiments.

**(F)** MKRN3-mediated ubiquitination impaired PABPC1-activated *GNRH1* (UTR) luciferase mRNA stability. *MKRN3^-/-^* HEK293T cells were co-transfected with vectors encoding *GNRH1* (UTR) luciferase, PRL-TK, MKRN3, and PABPC1 or PABPC1_4KR_, and treated with Actinomycin D at indicated time points. Total mRNAs were extracted, qPCR were done, and the half-lives of *Firefly* mRNA (t_1/2_) were calculated. The ubiquitination statuses of PABPC1 were also detected by immunoblotting in each group. Data were presented as Mean ± SD, three independent experiments.

**(G)** MKRN3 repressed PABPC3-activated *GNRH1*(UTR) luciferase activity. *MKRN3^-/-^* HEK293T cells were co-transfected with *GNRH1*(UTR) luciferase reporter as well as plasmids expressing PABPC3, and MKRN3 or MKRN3(C340G). Data were presented as Mean ± SD, one-way ANOVA with Bonferroni post-hoc test. **P* < 0.05, significant difference; ***P* < 0.01, very significant difference, three independent experiments.

**(H)** MKRN3 repressed PABPC4-activated *GNRH1*(UTR) luciferase activity. *MKRN3^-/-^* HEK293T cells were co-transfected with the *GNRH1*(UTR) luciferase reporter as well as plasmids expressing PABPC4, and MKRN3 or MKRN3(C340G). Data were presented as Mean ± SD, one-way ANOVA with Bonferroni post-hoc test. ***P* < 0.01, very significant difference, three independent experiments.

**(I)** MKRN3-mediated ubiquitination impaired PABPC1-activated *Ripor2* and *Npepl1* mRNA stability. GT1-7 cells were transfected with empty vector, or vector expressing MKRN3 or MKRN3 (C340G), combined with PABPC1 or not, and then treated with Actinomycin D at indicated time points. Total mRNAs were extracted, qPCR was done, and the half-lives of *Firefly* mRNA (t_1/2_) were calculated. Data were presented as Mean ± SD, three independent experiments.

**(J)** MKRN3 or PABPC1 has little effect on the stability of *Hist1h1e* and Hist1h2ah, which lack poly(A) tails. GT1-7 cells were transfected with empty vector, or vector expressing MKRN3 or MKRN3 (C340G), and then treated with Actinomycin D at indicated time points. Total mRNAs were extracted, qPCR was done, and the half-lives of *Firefly* mRNA (t_1/2_) were calculated. Data were presented as Mean ± SD, three independent experiments.

**(K)** MKRN3-mediated the ubiquitination of PABPC1 regulates the expression of endogenous GnRH1 at both mRNA and protein levels in GT1-7 cells, as revealed by qPCR or ELISA, respectively. GT1-7 cells were transfected with indicated plasmids, and the mRNA and protein levels of GnRH1 were examined 48 hrs later. Data were presented as Mean ± SD, one-way ANOVA with Bonferroni post-hoc test. ***P* < 0.01, very significant difference; NS, no significant difference, three independent experiments.

**Figure S6. The ubiquitination of PABPs by MKRN3 comprises its binding to the poly(A) of mRNA and the formation of translation initiation complex (TIC)**

**(A)** Test the specificity of PABPC1 and PABPC4 antibodies. Recombinant His6-tagged PABPC1 and PABPC4 were detected by immunoblotting using anti-His, anti-PABPC1 or anti-PABPC4 antibodies.

**(B)** More PABPC1 and PABPC4 proteins were recovered with poly(A)-tailed mRNAs in GT1-7 cells transfected with empty vector or that encoded MKRN3(C340G) than those encoded MKRN3, as revealed poly (U) pull-down assay.

**(C-D)** RNA competition assay was performed with recombinant His6 tagged PABPC1 (C) or PABPC4 (D) and γ-^32^P labeled (hot) RNA probes (A)_30_, with or without the unlabeled RNA oligomers (cold) of the same sequence (the hot-to-cold oligos were added at a 1:10 ratio).

**(E)** MKRN3-mediated the ubiquitination of PABPC4 compromised its binding to (A)_30_ RNA as detected by RNA EMSA. *MKRN3^-/-^* HEK293 cells were co-transfected with the indicated plasmids. 24 hours later, cell lysates were immunoprecipitated with anti-Flag affinity gels, treated with Usp2cc or not, and eluted with Flag peptides before subjected to RNA EMSA analysis.

**(F)** MKRN3-mediated ubiquitination compromised the affinity of PABPC4 to (A)_30_ RNA. EMSA assay was performed using (A)_30_ RNA probe that labeled with γ-^32^P. Recombinant PABPC4 was subjected to *in vitro* ubiquitination under varying indicated conditions, then immunoprecipitated with anti-Flag affinity gels before subjected to the EMSA assay.

**(G)** MKRN3 repressed the binding of endogenous PABPC1 to *Gnrh1* mRNAs in GT1-7 cells. RNA immunoprecipitation assay was performed with GT1-7 cells transfected with empty vector, MKRN3 or MKRN3(C340G), and the PABPC1-bound mRNAs were enriched with anti-PABPC1 antibody followed by qPCR assays using primers f1+r1(see in **Fig.6D**). Data were presented as Mean ± SD, one-way ANOVA with Bonferroni post-hoc test. ***P* < 0.01, of very significant difference, three independent experiments.
